# Supplementary material for: Downscaling epidemiological time series data for improving forecasting accuracy: An algorithmic approach
Source: PLoS One. 2023 Dec 14;18(12):e0295803. doi: 10.1371/journal.pone.0295803 (PMC10721108; doi:10.1371/journal.pone.0295803)
Supplement: Attachment — Submitted filename: PONE-D-23-27740.pdf [file pone.0295803.s004.pdf]

PLOS ONE

Downscaling Epidemiological Time Series Data for Improving Forecasting Accuracy: An Algorithmic Approach

--Manuscript Draft--

**Manuscript Number:**	PONE-D-23-27740
**Article Type:**	Research Article
**Full Title:**	Downscaling Epidemiological Time Series Data for Improving Forecasting Accuracy: An Algorithmic Approach
**Short Title:**	Downscaling Epidemiological Time Series Data for Improving Forecasting Accuracy
**Corresponding Author:**	Md. Kamrujjaman, Ph.D<br>University of Dhaka Faculty of Science<br>Dhaka, Dhaka District BANGLADESH
**Keywords:**	epidemiology; Downscaling Algorithm; Temporal Downscaling; ARIMA; Fourier-ARIMA; Dengue.
**Abstract:**	Data scarcity and discontinuity are common occurrences in the healthcare and epidemiological dataset and often need help forming an educative decision and forecasting the upcoming scenario. Often, these data are stored as monthly/yearly aggregate where the prevalent forecasting tools like Autoregressive Integrated Moving Average (ARIMA), Seasonal Autoregressive Integrated Moving Average (SARIMA), and TBATS often fail to provide satisfactory results. Artificial data synthesis methods have been proven to be a powerful tool for tackling these challenges. The paper aims to propose a downscaling data algorithm based on the underlying distribution. Our findings show that the synthesized data agrees with the original data regarding trend, seasonality, and residuals. The synthesized data provides a stable foothold for the forecasting tools to generate a more accurate forecast of the situation.
**Order of Authors:**	Mahadee Al Mobin, MS
	Md. Kamrujjaman, Ph.D
**Additional Information:**	
**Question**	**Response**
**Financial Disclosure**<br><br>Enter a financial disclosure statement that describes the sources of funding for the work included in this submission. Review the submission guidelines for detailed requirements. View published research articles from *PLOS ONE* for specific examples.<br><br>This statement is required for submission and **will appear in the published article** if the submission is accepted. Please make sure it is accurate.	The author(s) received no specific funding for this work.

Powered by Editorial Manager® and ProduXion Manager® from Aries Systems Corporation

**Unfunded studies**<br>Enter: *The author(s) received no specific funding for this work.*<br><br>**Funded studies**<br>Enter a statement with the following details:<br>• Initials of the authors who received each award<br>• Grant numbers awarded to each author<br>• The full name of each funder<br>• URL of each funder website<br>• Did the sponsors or funders play any role in the study design, data collection and analysis, decision to publish, or preparation of the manuscript?<br>• **NO** - Include this sentence at the end of your statement: *The funders had no role in study design, data collection and analysis, decision to publish, or preparation of the manuscript.*<br>• **YES** - Specify the role(s) played.<br><br>* typeset	
**Competing Interests**<br><br>Use the instructions below to enter a competing interest statement for this submission. On behalf of all authors, disclose any competing interests that could be perceived to bias this work—acknowledging all financial support and any other relevant financial or non-financial competing interests.<br><br>This statement is required for submission and **will appear in the published article** if the submission is accepted. Please make sure it is accurate and that any funding sources listed in your Funding Information later in the submission form are also declared in your Financial Disclosure statement.<br><br>View published research articles from *PLOS ONE* for specific examples.	The authors have declared that no competing interests exist.

Powered by Editorial Manager® and ProduXion Manager® from Aries Systems Corporation

**NO authors have competing interests**<br><br>Enter: *The authors have declared that no competing interests exist.*<br><br>**Authors with competing interests**<br><br>Enter competing interest details beginning with this statement:<br><br>*I have read the journal's policy and the authors of this manuscript have the following competing interests: [insert competing interests here]*<br><br>* typeset	
**Ethics Statement**<br><br>Enter an ethics statement for this submission. This statement is required if the study involved:<br><br>• Human participants<br>• Human specimens or tissue<br>• Vertebrate animals or cephalopods<br>• Vertebrate embryos or tissues<br>• Field research<br><br>Write "N/A" if the submission does not require an ethics statement.<br><br>General guidance is provided below. Consult the submission guidelines for detailed instructions. **Make sure that all information entered here is included in the Methods section of the manuscript.**	N/A

**Format for specific study types**<br><br>**Human Subject Research (involving human participants and/or tissue)**<br>• Give the name of the institutional review board or ethics committee that approved the study<br>• Include the approval number and/or a statement indicating approval of this research<br>• Indicate the form of consent obtained (written/oral) or the reason that consent was not obtained (e.g. the data were analyzed anonymously)<br><br>**Animal Research (involving vertebrate animals, embryos or tissues)**<br>• Provide the name of the Institutional Animal Care and Use Committee (IACUC) or other relevant ethics board that reviewed the study protocol, and indicate whether they approved this research or granted a formal waiver of ethical approval<br>• Include an approval number if one was obtained<br>• If the study involved *non-human primates*, add *additional details* about animal welfare and steps taken to ameliorate suffering<br>• If anesthesia, euthanasia, or any kind of animal sacrifice is part of the study, include briefly which substances and/or methods were applied<br><br>**Field Research**<br><br>Include the following details if this study involves the collection of plant, animal, or other materials from a natural setting:<br>• Field permit number<br>• Name of the institution or relevant body that granted permission	
**Data Availability**<br><br>Authors are required to make all data underlying the findings described fully available, without restriction, and from the time of publication. PLOS allows rare exceptions to address legal and ethical concerns. See the PLOS Data Policy and FAQ for detailed information.	No - some restrictions will apply

Powered by Editorial Manager® and ProduXion Manager® from Aries Systems Corporation

A Data Availability Statement describing where the data can be found is required at submission. Your answers to this question constitute the Data Availability Statement and **will be published in the article**, if accepted.<br><br>**Important:** Stating 'data available on request from the author' is not sufficient. If your data are only available upon request, select 'No' for the first question and explain your exceptional situation in the text box.<br><br>Do the authors confirm that all data underlying the findings described in their manuscript are fully available without restriction?	
**Describe where the data may be found in full sentences. If you are copying our sample text, replace any instances of XXX with the appropriate details.**<br><br>• If the data are **held or will be held in a public repository**, include URLs, accession numbers or DOIs. If this information will only be available after acceptance, indicate this by ticking the box below. For example: *All XXX files are available from the XXX database (accession number(s) XXX, XXX.)*.<br>• If the data are all contained **within the manuscript and/or Supporting Information files**, enter the following: *All relevant data are within the manuscript and its Supporting Information files.*<br>• If neither of these applies but you are able to provide **details of access elsewhere**, with or without limitations, please do so. For example:<br><br>*Data cannot be shared publicly because of [XXX]. Data are available from the XXX Institutional Data Access / Ethics Committee (contact via XXX) for researchers who meet the criteria for access to confidential data.*<br><br>*The data underlying the results presented in the study are available from (include the name of the third party*	1. Covid-19 data's are publicly available.<br>2. Data cannot be shared publicly because of Government policies. Data are available from the "Bangladesh Institute of Governance and Management (BIGM)" Institutional Data Access for researchers who meet the criteria for access to confidential data.<br>https://www.bigm.edu.bd/

and contact information or URL).<br>• This text is appropriate if the data are owned by a third party and authors do not have permission to share the data.<br><br>* typeset	
Additional data availability information:	

Manuscript

Click here to access/download;Manuscript;23_Manuscript_data_Sep.pdf

Downscaling Epidemiological Time Series Data for Improving Forecasting Accuracy: An Algorithmic Approach

Mahadee Al Mobin*[1,2] and Md. Kamrujjaman†[1]

[1]Department of Mathematics, University of Dhaka, Dhaka 1000, Bangladesh
[2]Bangladesh Institute of Governance and Management, Dhaka, Bangladesh

Abstract. Data scarcity and discontinuity are common occurrences in the healthcare and epidemiological dataset and often need help forming an educative decision and forecasting the upcoming scenario. Often, these data are stored as monthly/yearly aggregate where the prevalent forecasting tools like Autoregressive Integrated Moving Average (ARIMA), Seasonal Autoregressive Integrated Moving Average (SARIMA), and TBATS often fail to provide satisfactory results. Artificial data synthesis methods have been proven to be a powerful tool for tackling these challenges. The paper aims to propose a downscaling data algorithm based on the underlying distribution. Our findings show that the synthesized data agrees with the original data regarding trend, seasonality, and residuals. The synthesized data provides a stable foothold for the forecasting tools to generate a more accurate forecast of the situation.

Keywords: Epidemiology; Downscaling Algorithm; Temporal Downscaling; ARIMA; Fourier-ARIMA; Dengue.

Remark: The preprint of the manuscript was uploaded in **arxiv.org** and can be found in the link https://arxiv.org/abs/2304.12305. For more details-
Mobin, M. A., and Md. Kamrujjaman (2023): Downscaling Epidemiological Time Series Data for Improving Forecasting Accuracy: An Algorithmic Approach. arXiv preprint arXiv:2304.12305, https://doi.org/10.48550/arXiv.2304.12305.

Significance of this study and the importance of the results: Healthcare and epidemiological datasets often contain a limited amount of data and may have gaps, making it difficult to understand the underlying patterns or trends. These datasets are typically aggregated over a period, such as monthly or yearly summaries, which can also limit the ability to capture more granular insights. These challenges can make it difficult to make informed decisions or predict future scenarios. Traditional statistical forecasting models often fail to provide satisfactory results when the data is sparse or has a large number of gaps. To address this challenge, researchers have developed techniques to create artificial data that simulate the patterns in the original data. These techniques are known as artificial data synthesis methods. The goal of these methods is to generate data that is similar to the original dataset, in terms of its patterns, trends, and seasonality. The novel method proposed in the paper aims to generate synthetic data that matches the underlying distribution of the original data. This paper finds that the generated data was in agreement with the original data in terms of trend, seasonality, and residuals. This provides a stable foothold for the forecasting models to generate a much more accurate forecast of the situation. By using these

*Email: mahadeealmobin@gmail.com
†Corresponding author: Md Kamrujjaman, E-mail: kamrujjaman@du.ac.bd

artificial data synthesis methods, decision-makers can gain a better understanding of the underlying patterns and trends in the data. This can help them make more informed decisions and plan for the future. Additionally, these techniques can improve the accuracy of forecasting models, which can be critical in healthcare and epidemiological contexts, where accurate forecasting can mean the difference between life and death.

1 Introduction

Any process that involves deriving high-resolution data from low-resolution variables is referred to as downscaling. This method relies on dynamical or statistical approaches and is extensively utilized in the field of meteorology, climatology, and remote sensing [1, 2]. Significant exploration of the downscaling methods has been done in the field of geology and climatology to enhance the out of existing models like the General Circulation Model (GCM) [3–8], Regional Climate Model (RCM) [9], Integrated Grid Modeling System (IGMS) [10], System Advisor Model (SAM) [10] and to make it usable for the forecast of geographically significant region and time. Several methods has been used to downscale these data such as BCC/RCG-Weather Generators (BCC/RCG-WG) [11–13], and Statistics Downscaling Model (SDSM)[11, 14–19], Bayesian Model Averaging (BMA) [20]. Even machine learning methods has been used like Genetic algorithm (GA) [9], K Nearest Neighbourhood Resampling (KNNR) [9], Support Vector Machine (SVM) [11, 21–23]. Except for the machine learning algorithms, which are methods that are finding their applications in new domains, the rest of the methods are tailored to suit the outputs of the models, as mentioned earlier.

This class of methods has recently been applied in the disaggregation of spatial epidemiological data [24, 25]. Nevertheless, significant work has yet to be done for the temporal downscaling of epidemiological data. The temporal downscaling techniques are often classical interpolation techniques that do not do justice to aggregated data. This phenomenon can be well illustrated with an example. Consider the case of monthly Dengue infection data of 2017 from Figure 1, which has been downscaled using linear interpolation by considering the aggregated value as the value of the end date of a month in Figure 2. In this case, if we consider the monthly aggregate of the downscaled data, it does not match the original aggregate. This downscaled data, which differs from the original data in such statistical measures, shall result in decisions and knowledge that cannot be far from the truth.

This paper aims to propose a novel algorithm named Stochastic Bayesian Downscaling (SBD) algorithm based on the Bayesian approach that can regenerate downscaled temporal time series of varying time lengths from aggregated data preserving most of the statistical characteristics and the aggregated sum of the original data.

The paper is organized as follows. Section 2 describes the data used for the paper and its sources. Section 3 discusses the methodology at length with the proposed SBD algorithm. Section 4 compares the synthesized data with the actual data of two different epidemiological cases (Dengue and COVID-19) in Bangladesh and shows how the SBD algorithm could generate statistically accurate approximate of the actual with very little input in both cases discuss the benchmark metric used for evaluating the output. Section 5 shows the improvement of the forecasting accuracy using synthesized data over aggregated data using a statistical forecasting toolbox in the dengue scenario of Bangladesh using the last 12 years of monthly aggregated data, Forecasting model selection

procedures, and residuals. Finally, section 6 concludes our paper with an overview of the paper and how our paper has contributed to the existing literature and scopes for improvements and fields of application of the SBD algorithm.

2 Data

The dengue data from Bangladesh used in this paper are from January 2010 to July 2022 and are collected from DGHS [26], and IEDCR [27]. The COVID-19 data of Bangladesh are from 8 March 2020 to December 2020 and are collected from the WHO data repository [28].

3 Methodology

The SBD algorithm can be segmented into three sequential parts, as exhibited in Figure 3. Initially, the algorithm considers a prior distribution to generate synthetic downscaled data. The SBD algorithm considers the aggregated data as the prior distribution of the downscaled data. For example: If we have the monthly epidemiological data of dengue for the year 2017, thus to attain the prior distribution for the downscaled data, we divide the data by 30. The fact is well illustrated in Figures 1 and 2. Figure 1 depicts the monthly distribution of the DENV (Dengue Virus) infection in Bangladesh for the year 2017, and Figure 2 represents the prior distribution obtained by the method described above.

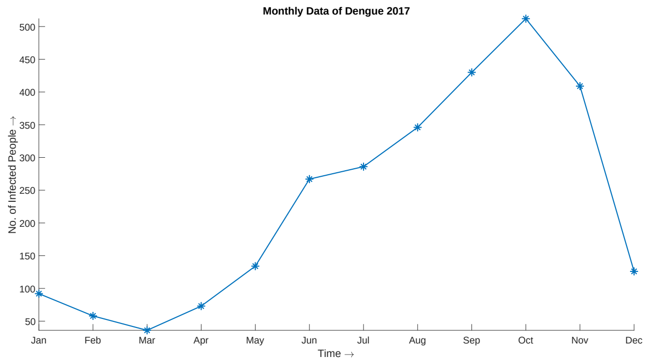

Figure 1: The monthly aggregate of the DENV infection in Bangladesh in the year 2017.

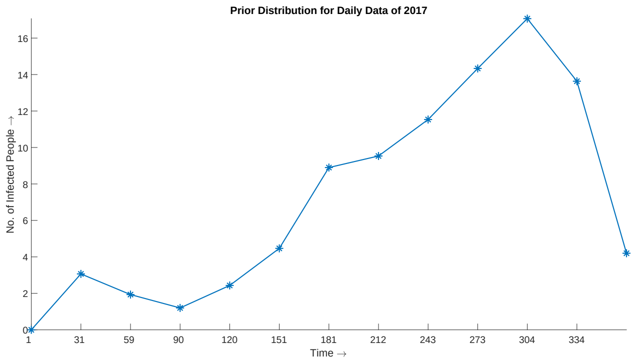

Figure 2: The monthly aggregate of the DENV infection in Bangladesh in the year 2017.

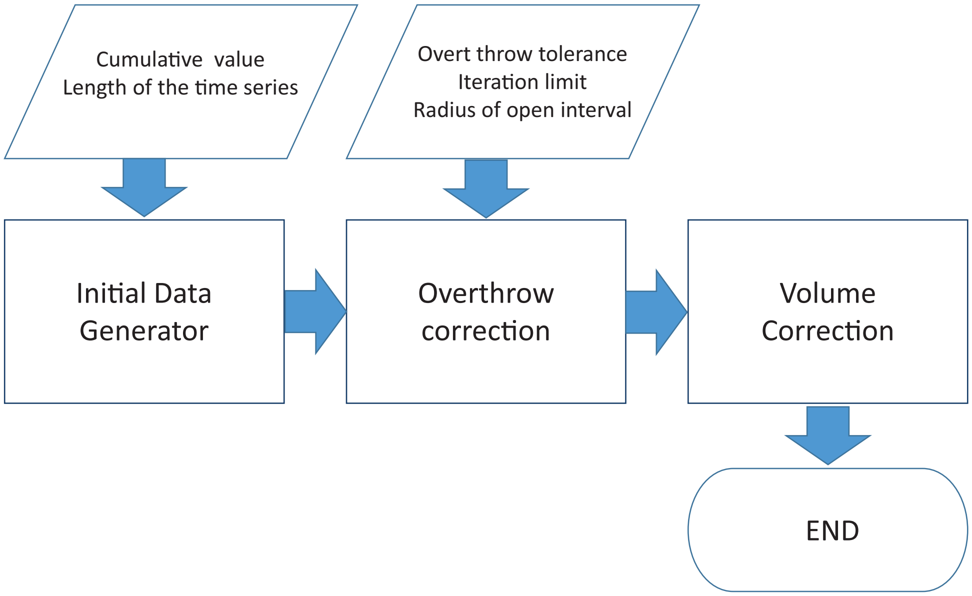

Figure 3: Flow diagram of the SBD algorithm.

Based on the prior distribution, initial statistical properties of the synthetic data are obtained except for the standard deviation (σ). As σ is scaling independent, hence scaling method used to obtain the prior distribution from the monthly aggregate keeps the σ identical to the monthly aggregate. To overcome this problem, we consider,

$$\sigma_0 = \frac{\sigma_{prior\,distribution}}{30} \tag{3.1}$$

where σ_0 is the standard deviation considered for the distribution to be fitted to generate the downscaled data by the algorithm and $\sigma_{prior\,distribution}$ is the standard deviation of the obtained prior distribution. Later on, in section 4, we will see that the initial assumption of the standard deviation considered in (3.1) is a good approximation for the downscaled data.

3.1 Initial Data Generation

The *"Initial Data Generator"* phase feeds on the aggregated data, length of the aggregate interval, and σ_0 to give an initial downscaled data based on a "Distribution Generator". Based on the prior distribution, a proper statistical probability distribution (PD) is to be considered to be fitted to generate the data. The "Distribution Generator" aims to fit the selected PD to the prior distribution based on the statistical properties obtained for the initial phase. The challenge in this scenario and every step of the algorithm is ensuring that the synthetic data produced in every step is non-negative integers, as we are dealing with epidemiological data. Thus specific measures have been deployed to tackle these challenges, which are:

- To ensure non negativity consider the transformation:

$$\hat{\mathbf{y}} = \mathbf{y} + min(|\mathbf{y}|)$$

- To ensure that the data points are integer irrespective of the selection of PD, we round off the data to the nearest integer and subtract one from randomly selected data points in each aggregated unit such that the synthesized data has the same sum as the aggregated unit

Thus imposing these measures, the "Distribution Generator" generates synthetic distribution for each aggregated unit. Thus, looping over the entire aggregated timeline generates the initial distribution of the downscaled data concerning the aggregated data. This initial distribution is a suitable approximation to the actual data but can be improved with further refinement. The synthetic data will result in the exact aggregated data from which it is generated.

3.2 Overthrow Correction

This step is often necessary for time series data with an abrupt change in gradient or in case of initial approximation with abnormally large overthrow as the approximations are probabilistic. In case of data with the abrupt change in gradient, the initial approximation is often left with a staircase-like structure as exhibited in the Figure 4. The problem can be corrected using the overthrow correction measure, which is demonstrated in Figure 5.

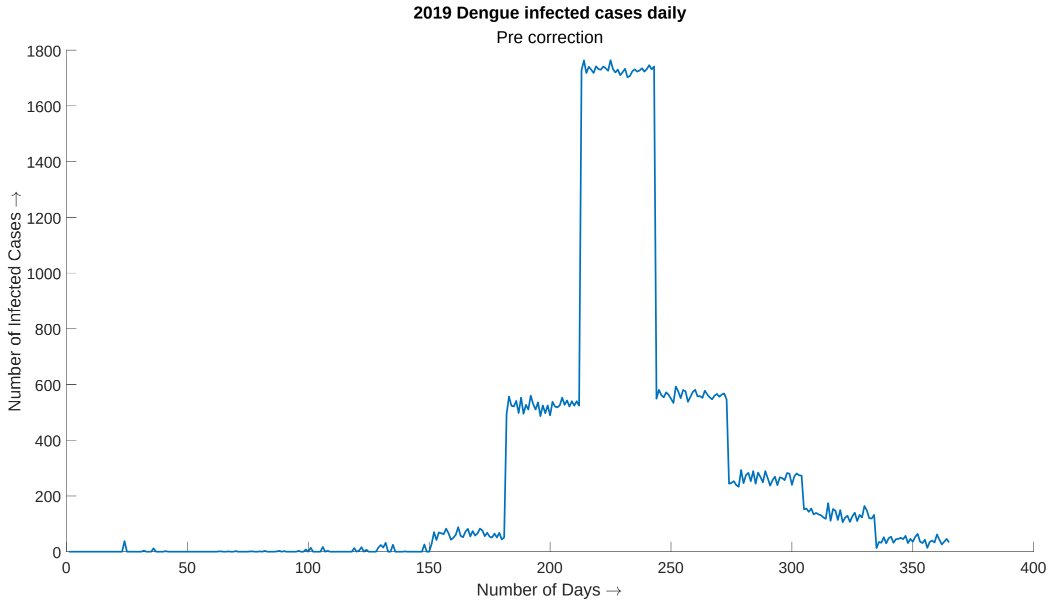

Figure 4: Initial approximation without overthrow correction exhibits a staircase like property due to higher gradient change of the prior distribution.

The overthrow correction part takes a tolerance, δ, iteration limit, n, and a radius of an open interval, r. The step initially determines overthrow using tolerance between two neighboring points, i.e., if $y_i - y_{i-1} > \delta$ or $y_i - y_{i+1} > \delta$ then y_i is an overthrow. After identifying an overthrow, we consider an open interval of radius r around the overthrow point and execute the distribution generator on that open interval. This redistributes the sample within the open interval diminishing the overthrow to some extent. This process is iterated n times over the entire time series to ensure satisfactory results. The strength of the overthrow correction step can be dictated by the two parameters δ and n. The strength of the overthrow correction is directly proportional to n and is inversely proportional to δ. Selecting the correct parameter value can ensure a good approximation of the real-life scenario.

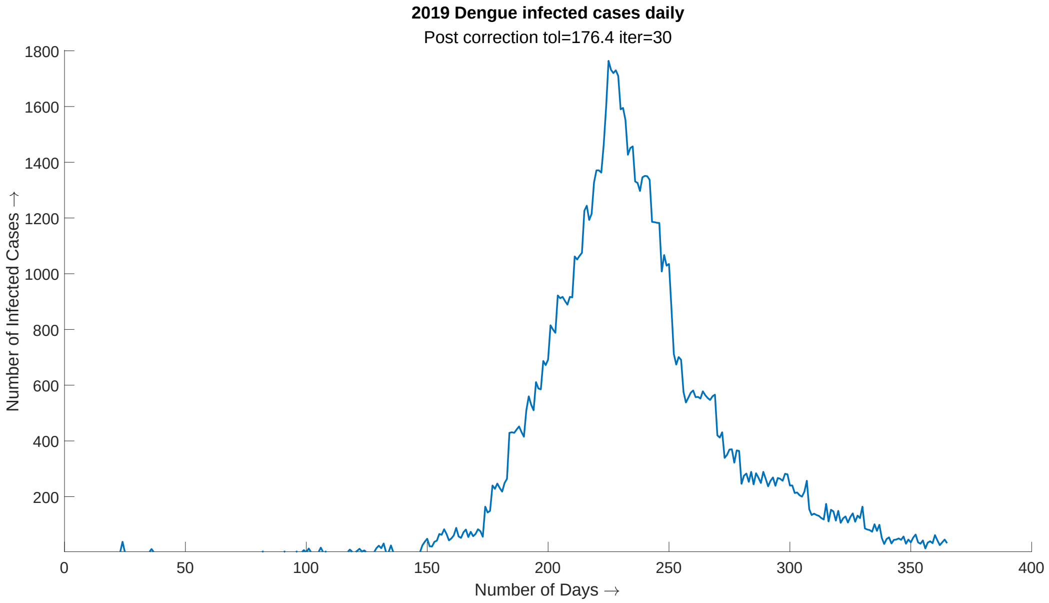

Figure 5: Initial approximation with overthrow correction exhibits a much proper approximation of the real case scenario preserving its original trend.

3.3 Volume Correction

The overthrow correction disrupts the property of the synthesized time series to conserve its aggregated sum equal to the given aggregated distribution due to its local correction property. The scenario best illustrates the table 1. This problem is addressed in this step. To maintain aggregated sum equal to the original data, we consider each aggregated unit and adjust the sum accordingly, adding/subtracting 1 from randomly chosen indices until the sum equates as required.

Month	Actual	Initial Distribution	Overthrow Correction	Volume Correction
January	38	38	42	38
February	18	18	13	18
March	17	17	18	17
April	58	58	61	58
May	193	193	300	193
June	1884	1884	2500	1884
July	16253	16253	17617	16253
August	53636	53636	49581	53636
September	16856	16856	18259	16856
October	8143	8143	8419	8143
November	4011	4011	4094	4011
December	1247	1247	1450	1247
**Total**	**102354**	**102354**	**102354**	**102354**

Table 1: The table exhibits the comparison of the number of cases each month for executing the SBD algorithm on the Dengue 2019 data of Bangladesh with the actual data. Here we can see the total number of infected individuals in each algorithm step is the same. In the case of the monthly sum, we see some anomaly in the overthrow correction case, which has been fixed in the volume correction step.

3.4 The Stochastic Bayesian Downscaling (SBD) Algorithm

The algorithm calls for a unique name. From now on, we shall address it as Stochastic Bayesian Downscaling (SBD) algorithm. The structural part of the algorithm has been discussed at length in the first three subsections of the section methodology. The proper pseudo code of the SBD algorithm is as follows:

Algorithm 1: Stochastic Bayesian Downscaling (SBD) Algorithm

Require: Aggregated value vector, **v**
  Overthrow tolerance, δ
  Iteration limit, n
  Radius of the open interval, r
  Standard deviation, σ
Ensure: downscaled time series, $\bar{v}$
  for elem in **v** **do**
    $\bar{v}$ = Distribution Generator(elem,σ)
  end for
  for i from 1 to n **do**
    find a vector of coordinates of overthrow points
    for elem in overthrow points **do**
      open interval centering elem of radius, r = Distribution Generator(sum of the elements of open interval,σ)
    end for
  end for

for elem in **v** **do**
  if $v_i \neq$sum of euiquivalent aggregate in $\bar{v}$ **then**
    d=v_i-sum of equivalent aggregate in $\bar{v}$
    while $d \neq 0$ **do**
      if $d > 0$ **then**
        $\bar{v}_{randomly\,picked\,index} = \bar{v}_{randomly\,picked\,index} + 1$
        $d- = 1$
      else
        $\bar{v}_{randomly\,picked\,index} = \bar{v}_{randomly\,picked\,index} - 1$
        $d- = 1$
      end if
    end while
  end if
end for

Algorithm 2: Distribution Generator

Require: Total sum of the down scaled distribution, s
  Standard deviation, σ
Ensure: Down scaled approximation over the length of the aggregate, $\bar{v}$
  $\bar{v}$ =Fit the decided distrubiton to the given down scaled time frame
  if elems in $\bar{v} < 0$ **then**
    $\bar{v} = \bar{v} + |min(\bar{v})|$
  end if
  if elems in $\bar{v}$ are not integer **then**
    $\bar{v} = round(\bar{v})$
  end if
  if $s \neq \sum \bar{v}$ **then**
    d=$s - \sum \bar{v}$
    while $d \neq 0$ **do**
      if $d > 0$ **then**
        $\bar{v}_{randomly\,picked\,index} = \bar{v}_{randomly\,picked\,index} + 1$
        $d- = 1$
      else
        $\bar{v}_{randomly\,picked\,index} = \bar{v}_{randomly\,picked\,index} - 1$
        $d- = 1$
      end if
    end while
  end if

The SBD algorithm is heavily dependent on the random selection of numbers that are prone to generate non-reproducible results. Thus seeding the random number generator is highly recom-

mended to ensure reproducible results.

The novelty of SBD algorithm is its consideration of the prior distribution as initialization and deploying the underlying distribution to generate synthesized downscaled data, which is non-negative and conserves the aggregated value of the given data.

4 Comparison of the Synthesized Data with the Real Data

To determine the accuracy of the SBD algorithm, we test the SBD algorithm against some real-world data. Here, we have taken 2020 COVID-19 data on infected individuals in Bangladesh and 2022 (January to July), Dengue data on infected individuals in Bangladesh. The data, as mentioned earlier, are daily data on the number of newly infected individuals nationwide. We aim to convert this data to monthly aggregate and feed the aggregated data to the algorithm to generate downscaled daily data; hence we can compare the accuracy of the synthetic daily data with the actual daily data. To determine the accuracy of the approximation, we will use two error measures and do component analysis on the real and synthetic data to see if the synthetic data can well approximate the underlying properties of the real data. In case of the component decomposition, we will use the additive model mentioned in (4.1),

$$y_i = Trend + Seasonality + Residual \tag{4.1}$$

as the procured data has some zero values for which the multiplicative model mentioned in (4.2)

$$y_i = Trend \times Seasonality \times Residual \tag{4.2}$$

is not suitable in this scenario.

4.1 Error Measures for Benchmark

To compare the result with the real world data we shall use two error terms that describes the overall error of the approximation. These are as follows:

- **Root Mean Square Error:**
  The root-mean-square deviation (RMSD) or root-mean-square error (RMSE) is a commonly used measure of the discrepancies between the values (sample or population values) predicted by a model or estimator and the actual values. RMSD is the square root of the second sample moment of the discrepancies between anticipated and observed values or the quadratic mean of these differences. When the computations are executed over the data set used for the estimate, these deviations are known as residuals. When computed out-of-sample, they are known as errors (or prediction errors). The RMSD aggregates the magnitudes of the mistakes in predictions for various pieces of data into a single metric of predictive ability. RMSD is a measure of accuracy used to assess the predicting losses of various models for a specific dataset and not between datasets because it is scale-dependent [29].

  RMSD is always positive, and a value of 0 would suggest a perfect fit to the data, which is nearly never attained in practice. A smaller RMSD is often preferable to a greater one. However, because the metric depends on the numbers' magnitude, comparisons between various kinds of data are invalid.

The root square of the mean of squared mistakes is the RMSD. The influence of each inaccuracy on RMSD is proportional to the magnitude of the squared error; therefore, more significant errors have an outsized effect on RMSD. As a result, the RMSD is extremely sensitive to outliers [30, 31].

Instead of the Root Mean Square Deviation, several scholars have suggested the Mean Absolute Error (MAE) as a useful statistical tool. The MAE has certain advantages over the RMSD when it comes to interpretability. The mean absolute error, abbreviated as MAE, is the average of the absolute values of the mistakes. The square root of the average of squared errors is more challenging to grasp than the MAE, simplifying things considerably. In addition, the magnitude of a mistake has an effect on the MAE in direct proportion to its absolute value, whereas the RMSD does not follow this pattern at all [30].

RMSE can be defined using the following formula:

$$\text{RMSE}=\sqrt{\frac{\sum_{i=1}^{N}(x_i-\hat{x}_i)}{N}}$$

where, x_i is the actual data and $\hat{x}_i$ is the predicted data.

- **Mass Absolute Error:**
  In statistics, the term "mean absolute error" (MAE) refers to a measurement of the errors that occur when matched observations expressing the same event are compared. Comparisons of what was predicted vs what was actually observed, subsequent time versus beginning time, and one technique of measurement versus an alternate technique of measurement are all examples of Y versus X. The mean absolute error (MAE) is determined by taking the sum of all absolute errors and dividing it by the total number of samples:

$$\text{MAE}=\frac{\sum_{i=1}^{N}|x_i-\hat{x}_i|}{N}$$

Therefore, it is an arithmetic average of the absolute errors, which may be represented as $|e_i| = |x_i - \hat{x}_i|$, where $\hat{x}_i$ represents the forecast and x_i represents the actual value. It is important to keep in mind that different formulations could use relative frequencies as weight factors. The scale that is being used to measure the data is also used for the mean absolute error. Because this is what's known as a scale-dependent accuracy measure, it can't be used to compare series that have different scales because the comparisons wouldn't be valid [32]. In time series analysis, the mean absolute error is a frequent way to quantify the accuracy of forecasts [29], occasionally leading to confusion with the more traditional definition of mean absolute deviation. There is, more generally speaking, the same confusion.

One of the many methods that may be used to compare forecasts with the transpired results is the mean absolute error. The mean absolute scaled error, also known as MASE, and the mean squared error are two options with a solid track record. The mean signed difference is one metric that does emphasize this, as opposed to the other measures, which all summarize performance in a fashion that disregards the direction of whether the forecast was made too

high or too low.

When fitting a prediction model with a chosen performance metric, the equivalent for mean absolute error is mean absolute deviations, and the least squares approach is related to the mean squared error.

Although some academics report and interpret it that way, mean absolute error (MAE) and root-mean-square error (RMSE) are not the same concepts. The MAE is conceptually more straightforward to perceive than the RMSE. It is the average absolute vertical or horizontal distance between each point in a scatter plot and the Y=X line. In contrast, the RMSE is a measure of error that is more difficult to interpret. To phrase this another way, MAE refers to the average absolute difference between X and Y. In addition, each error's contribution to the MAE is weighted according to the absolute value of the error. This is in contrast to the RMSE, which involves quadrupling the differences; as a result, a few significant changes will have a higher impact on the RMSE than they will have on the MAE [30].

Since many of the data points in the actual and synthesized cases is popluated with 0 hence Mass Absolute Percentage Error (MAPE), and Scaled Mass Absolute Percentage Error (MAPE) are undefined in this scenario.

4.2 Dengue

4.2.1 Preprocessing and Result

In case of this simulation, we took Bangladesh's 2022 daily Dengue infected data from January to July. To feed this data into the SBD algorithm, we convert the daily data to monthly aggregate as illustrated in Figure 6,

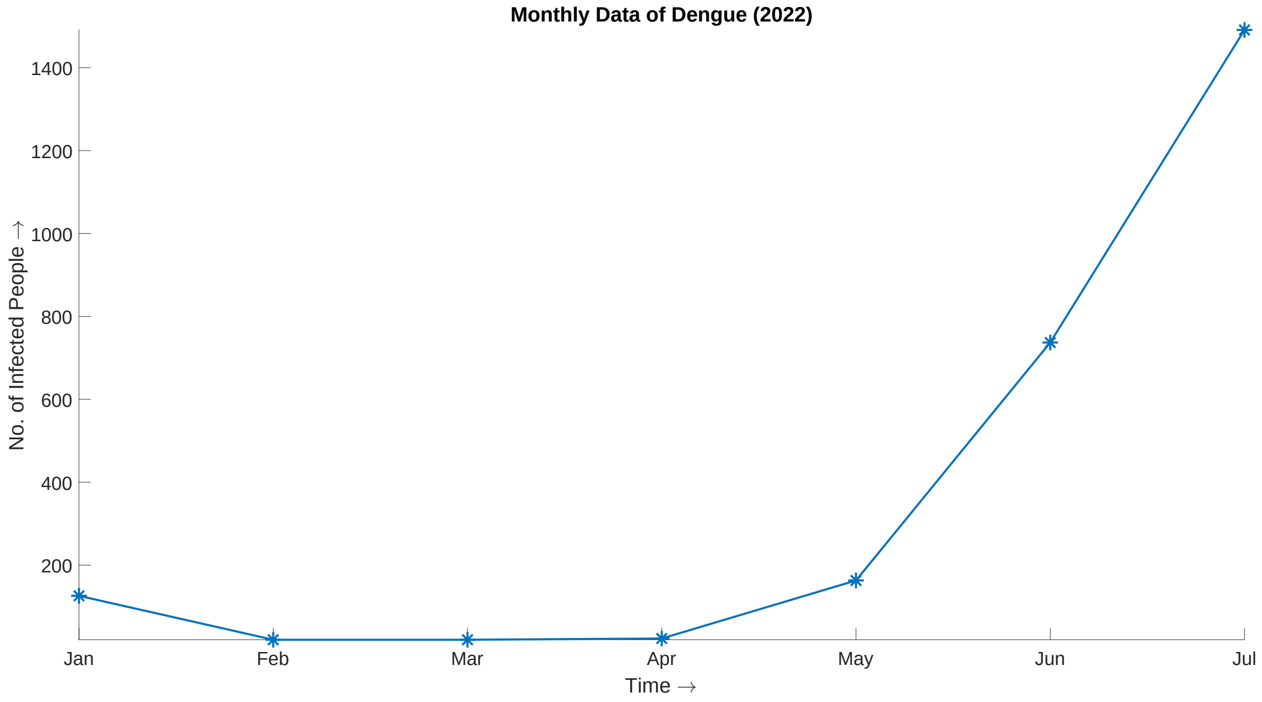

Figure 6: Monthly aggregate of 2022 Dengue data from January to July.

We feed in this data considering,

- Initial standard deviation, $\sigma_0 = \dfrac{\sigma_{prior\ distribution}}{30} = \dfrac{556.6431703}{30} = 18.55477234.$
- Over throw tolerance, $\delta = 0.6\times$ (Range of the initial distribution).
- Iteration limit, $n = 100$.
- Radius of open interval, $r = 3$.
- Underlying distribution to be normal.

and generate the synthesized data. Figure 8 illustrates the synthesized data, which can be said to be a good approximation of the actual given the aggregated prior distribution.

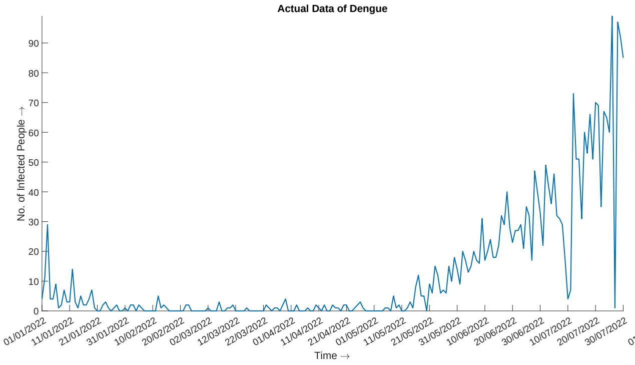

Figure 7: Daily number of infected cases of Dengue in 2022 from January to July collected from DGHS.

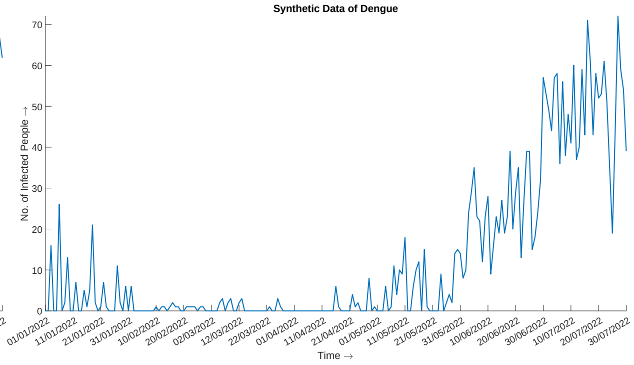

Figure 8: Synthesized daily number of infected cases of Dengue in 2022 from January to July.

4.2.2 Error Metrics and Statistical Measures

The calculated error measures are:

- $MAE = 6.60664$, which implies that the average error between the actual and synthesized data is 6.60664.
- $RMSE = 12.64499$ which implies that the standard deviation of the residuals/errors is 12.64499. The fact is well illustrated in Figure 14.

The error metric shows satisfactory results. The following table validates if the synthesized data honours the aggregated sum of the prior distribution.

**Month**	**Actual**	**Initial Distribution**	**Overthrow Correction**	**Volume Correction**
January	126	126	119	126
February	20	20	27	20
March	20	20	20	20
April	23	23	32	23
May	163	163	206	163
June	737	737	733	737
July	1491	1491	1443	1491
**Total**	**2580**	**2580**	**2580**	**2580**

Table 2: This table illustrates that the synthetic data agrees with the monthly sum of the actual data

The total number of cases in each scenario has been maintained equally. As discussed earlier, we can see that the initial distribution holds the monthly sum consistently, which gets disrupted in the overthrow correction phase and later corrected in the volume correction phase.

We shall now explore the basic statistical properties of the synthetic data with respect to the actual data.

**Measures**	**Observed**	**Synthesized**
Mean	12.22748815	12.22748815
Standard Deviation	20.28993189	18.49672823
Minimum	0	0
Lower Quartile(Q1)	0	0
Median	2	1
Upper Quartile(Q2)	17	19
Maximum	99	72

Table 3: This table illustrates the comparison of the basic statistical measures of the synthesized data with respect to the actual data.

It is to be noted that the mean of the synthesized data equates to that of the original data, although it was not plugged into the SBD algorithm in any manner. As previously discussed that σ_0 is a good approximation to the original σ. All the rest of the measures are somewhat close, but the maximum varies by a lot. The maximum is hard to anticipate from the aggregated data; hence it is an avenue that demands further exploration.

4.2.3 Component Decomposition and Comparison

We now want to do component decomposition of both the actual and synthetic data based on the model mentioned in (4.1). However, component decomposition is no benchmark for accuracy, but SBD algorithm aims to improve the outcome of forecasting techniques highly influenced by the components within a time series data. Thus comparing these components can answer the question of whether the components-based characteristics of the original time series are present within the synthesized data.

In the case of the trend component (Figure 9 and 10), both the actual and the synthesized data shows similar result and trend of the actual data have been well approximated by the trend of the synthesized data.

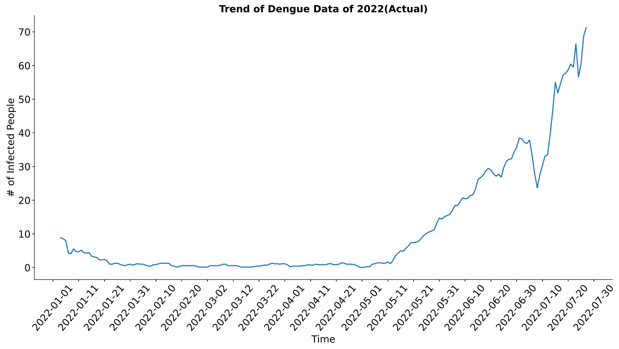

Figure 9: Trend of the actual dengue data.

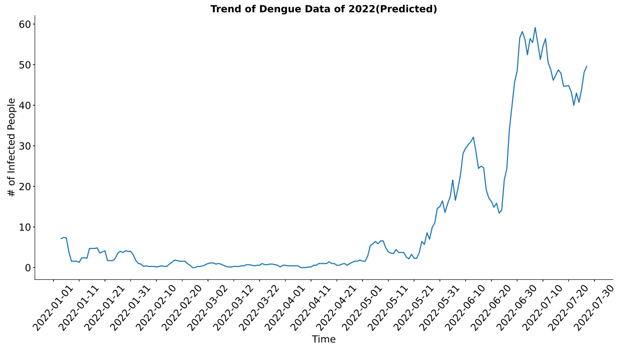

Figure 10: Trend of the synthetic dengue data.

In the case of the seasonality component (Figure 11 and 12), both the actual and the synthesized data show major weekly and minor sub-weekly seasonality. The synthesized data's seasonality approximates the actual data's seasonality well.

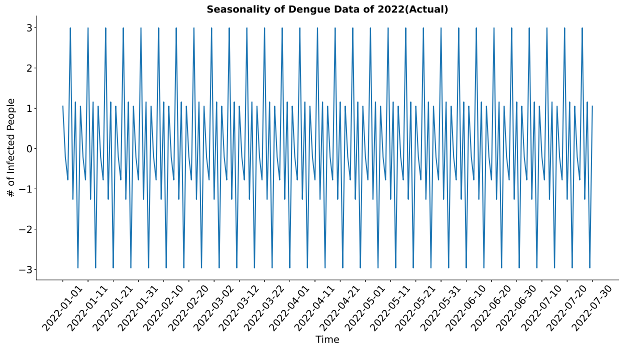

Figure 11: Seasonality of the actual dengue data.

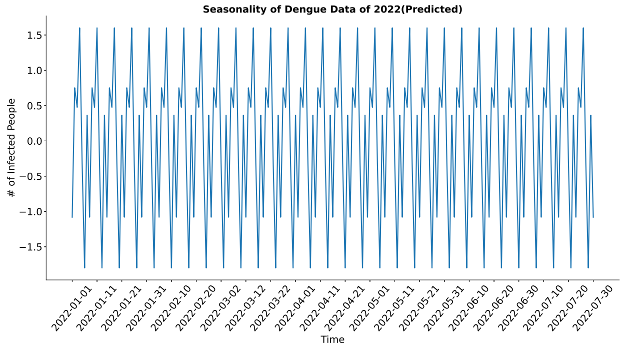

Figure 12: Seasonality of the synthetic dengue data.

In the case of the residual component (Figure 13 and 14), both the actual and the synthesized data show a similar result, although the residual of the synthetic data may look noisy at first glance but upon closer inspection, it is evident that the residual of the synthetic data shows less deviation from the standard value in comparison to the actual data. The synthesized data's residual has well approximated the actual data's residual.

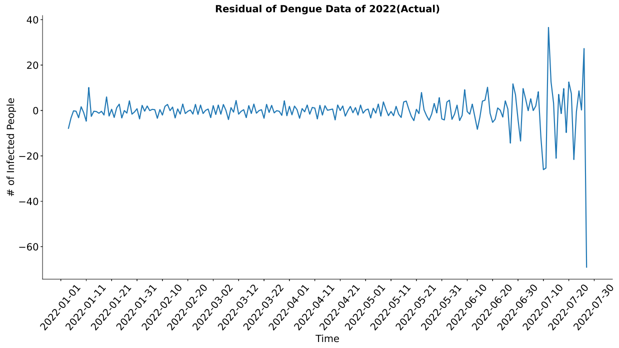

Figure 13: Residual of the actual dengue data.

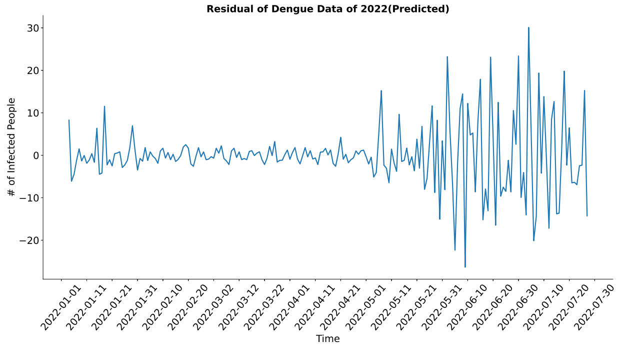

Figure 14: Residual of the synthetic dengue data.

As mentioned earlier, the key takeaway from the discussion is that the SBD algorithm could

generate an excellent approximation of the dengue data from the monthly aggregated data based on some statistical properties of the prior distribution. In the following section, we shall also test SBD algorithm's efficacy in another epidemiological scenario.

4.3 COVID-19

4.3.1 Preprocessing and Result

In case of this simulation, we took Bangladesh's 2020 daily COVID-19 infected data from March to December [33, 34]. To feed this data into the SBD algorithm, we convert the daily data to monthly aggregate as illustrated in Figure 15,

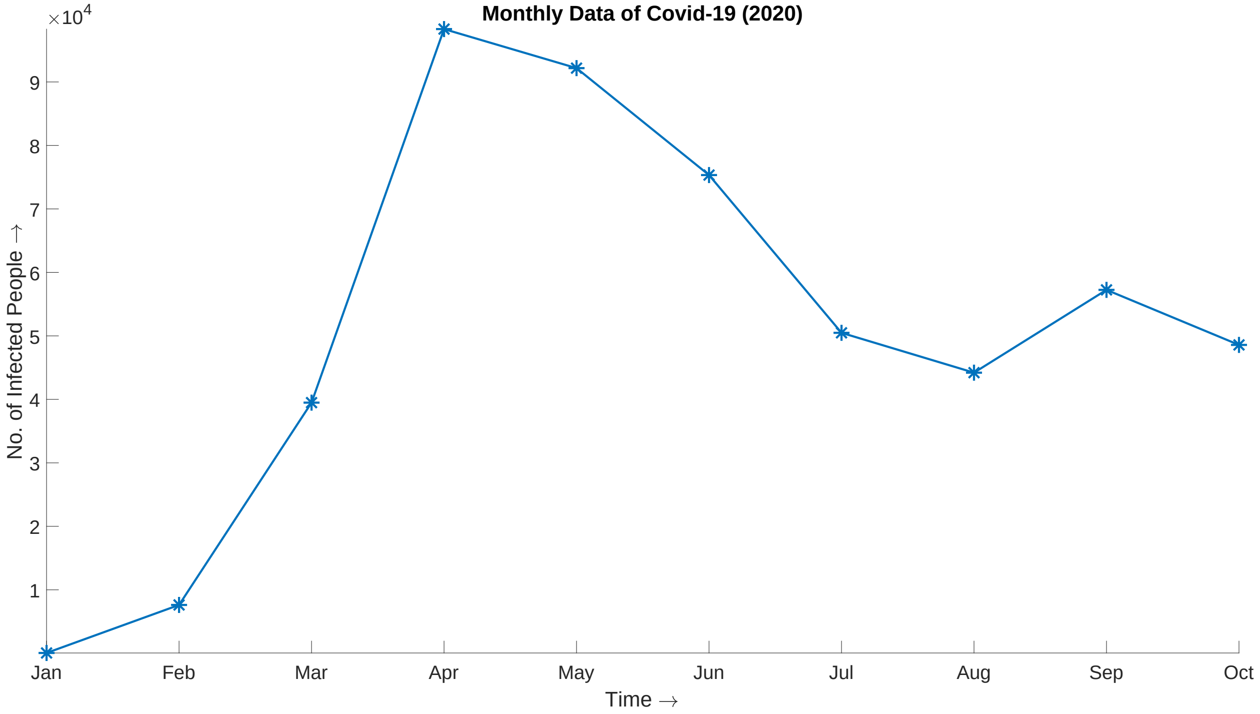

Figure 15: Monthly aggregate of 2020 COVID-19 infected data of Bangladesh from March to December.

We feed in this data considering,

- Initial standard deviation, $\sigma_0 = \dfrac{\sigma_{prior\,distribution}}{30} = \dfrac{32021.87439}{30} = 1067.395813.$
- Over throw tolerance, $\delta = 0.2\times$ (Range of the initial distribution).
- Iteration limit, $n = 100$.
- Radius of open interval, $r = 3$.
- Underlying distribution to be normal.

and generate the synthesized data. Figure 17 illustrates the synthesized data, which can be said to be a good approximation of the actual given the aggregated prior distribution.

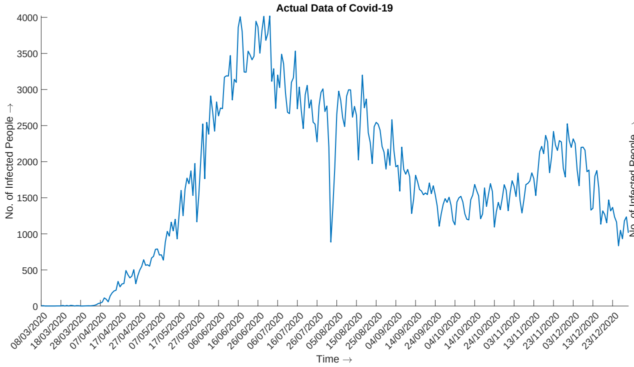

Figure 16: Daily number of infected cases of COVID-19 in 2020 from March to December collected from DGHS.

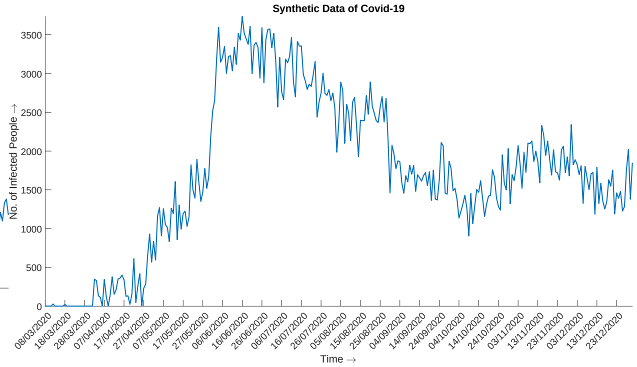

Figure 17: Synthesized daily number of infected cases of COVID-19 in 2020 from March to December.

4.3.2 Error Metrics and Statistical Measures

The calculated error measures are:

- $MAE = 257.41806$, which implies that the average error between the actual and synthesized data is 257.41806, which is reasonable considering the mean of the data is 1717.424749.
- $RMSE = 346.6241$, which implies that the standard deviation of the residuals/errors is 346.6241. The fact is well illustrated in Figure 23.

it is to be noted that the error term of this scenario must not be compared with the error term of the previous case as they are of varying scale. Compared to the scale of the data, the error metric shows satisfactory results. The following table validates if the synthesized data honours the aggregated sum of the prior distribution.

Month	Actual	Initial Distribution	Overthrow Correction	Volume Correction
March	51	51	51	51
April	7616	7616	9226	7616
May	39486	39486	41261	39486
June	98330	98330	94075	98330
July	92178	92178	92115	92178
August	75335	75335	75605	75335
September	50483	50483	50766	50483
October	44205	44205	45126	44205
November	57248	57248	55805	57248
December	48578	48578	49480	48578
**Total**	**513510**	**513510**	**513510**	**513510**

Table 4: This table illustrates that the synthetic data agrees with the monthly sum of the actual data.

The total number of cases in each scenario has been maintained equally. As discussed earlier, we can see that the initial distribution holds the monthly sum consistently, which gets a little disrupted

in the overthrow correction phase and is later on corrected in the volume correction phase.

We shall now explore the basic statistical properties of the synthetic data with respect to the actual data.

Measures	Observed	Synthesized
Count	299	299
Mean	1717.424749	1717.424749
Standard Deviation	1044.457258	1007.554237
Minimum	0	0
Lower Quartile(Q1)	1115.5	1225
Median	1666	1696
Upper Quartile(Q2)	2521.5	2481.5
Maximum	4019	3735

Table 5: This table illustrates the comparison of the basic statistical measures of the synthesized data with respect to the actual data.

It is to be noted that the mean of the synthesized data equates to that of the original data, although it was not plugged into the SBD algorithm in any manner. As previously discussed that σ_0 is a good approximation to the original σ. All the rest of the measures are somewhat close, but the maximum varies by a lot. The maximum is hard to anticipate from the aggregated data; hence it is an avenue that demands further exploration.

4.3.3 Component Decomposition and Comparison

We now want to do component decomposition of both the actual and synthetic data based on the model mentioned in (4.1). However, component decomposition in no way is a benchmark for accuracy, but as SBD algorithm aims to improve the outcome of forecasting techniques which are highly influenced by the components within a time series data. Thus, comparing these components can answer the question of whether the original time series's components-based characteristics are present in the synthesized data.

In case of the trend component (Figure 18 and 19) both the actual and the synthesized data shows similar result and trend of the actual data have been well approximated by the trend of the synthesized data.

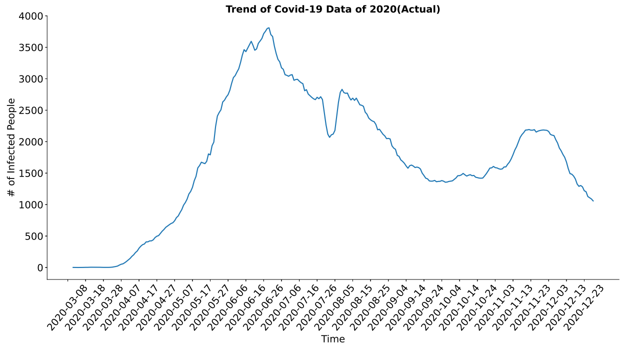

Figure 18: Trend of the actual COVID-19 data.

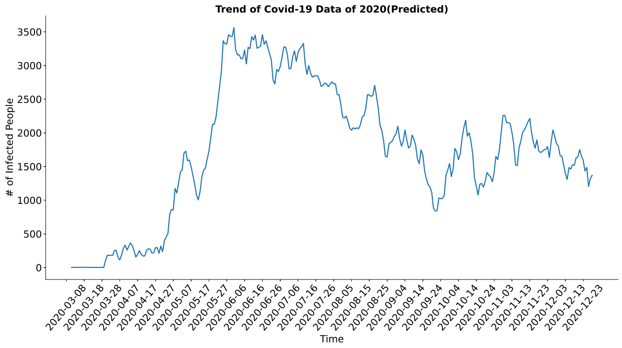

Figure 19: Trend of the synthetic COVID-19 data.

In case of the seasonality component (Figure 20 and 21), both the actual and the synthesized data shows major weekly seasonality. The seasonality of the synthesized data has well approximated the seasonality of the actual data.

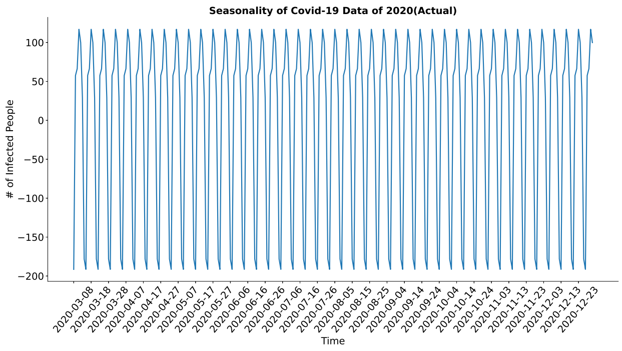

Figure 20: Seasonality of the actual COVID-19 data.

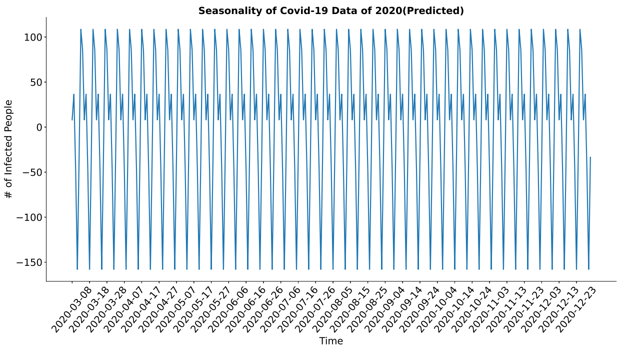

Figure 21: Seasonality of the synthetic COVID-19 data.

In the case of the residual component (Figure 22 and 23), both the actual and the synthesized data shows a similar result, although the residual of the synthetic data may look a bit noisy at first glance but upon closer inspection, it is evident that the residual of the synthetic data shows less deviation from the standard value in comparison to the actual data. The residual of the synthesized data has well approximated the residual of the actual data.

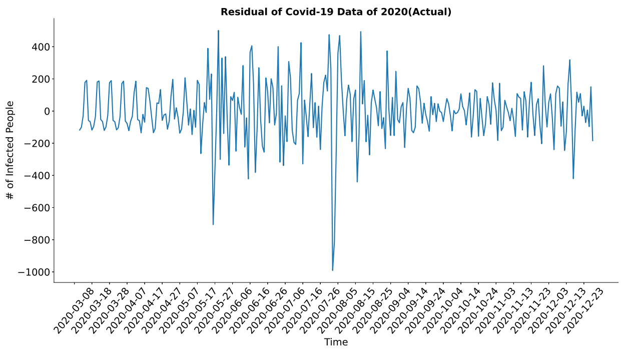

Figure 22: Residual of the actual dengue data.

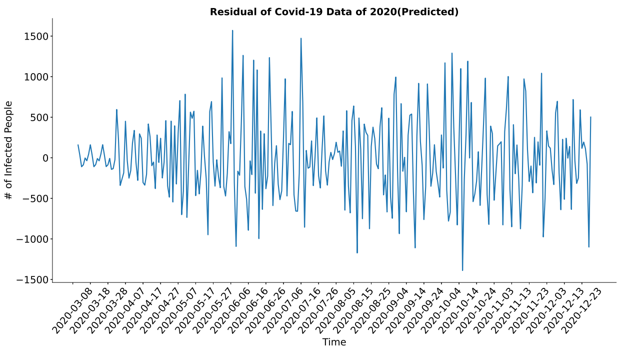

Figure 23: Residual of the synthetic COVID-19 data.

The key takeaway from the discussion above is that the algorithm could generate an excellent approximation of the COVID-19 data from the monthly aggregated data based on some statistical properties of the prior distribution. We shall also test SBD algorithm's efficacy in a forecasting scenario in the following section.

5 Improvements in Forecasting Accuracy

In this section, we shall forecast the Dengue infection case in Bangladesh using statistical forecasting tools. The use of statistical modelling is one of the helpful ways that may be utilized for the forecasting of dengue outbreaks [35, 36]. Previous research carried out in China [37], India [38], Thailand [39], West Indies [40], Colombia [41], Brazil [42], and Australia [43] on infectious diseases made substantial use of the time series technique in the field of epidemiologic research on infectious diseases [43]. A number of earlier research looked at the Autoregressive Integrated Moving Average

(ARIMA) model as a potential tool for use in forecasting [44, 45, 45, 45–47, 47, 48]. In addition, the ARIMA models have seen widespread use for dengue forecasting [47, 49–51]. When establishing statistical forecasting models, these are frequently paired with Seasonal Auto-regressive Integrated Moving Average (SARIMA) models, which have proven to be suitable for assessing time series data with ordinary or seasonal patterns [38, 40, 42, 43, 52]. It is likely that developing a dengue incidence forecasting model based on knowledge from previous outbreaks and environment variables might be an extremely helpful tool for anticipating the severity and frequency of potential epidemics.

ARIMA is a well-known model in statistics that is predominantly used to forecast and analyze time series data [53]. Auto Regression of order p can be defined as

$$Y_t = e_t + \sum_{i=1}^{p} \alpha_i Y_{t-i} \tag{5.1}$$

where e_t are white noises of mean 0 and variance σ_e^2.
The Moving Average (MA) of order q is defined as,

$$\hat{Y}_t = e_t + \sum_{i=1}^{q} \beta_i e_{t-i} \tag{5.2}$$

ARMA model in theory is formed in unison of (5.1) and (5.2). Hence, an ARMA model of order $(p,\ q)$ is defined

$$\hat{Y}_t = e_t + \sum_{i=1}^{p} \alpha_i Y_{t-i} + \sum_{i=1}^{q} \beta_i e_{t-i} \tag{5.3}$$

where the p and q are the corresponding order of the AR and MA. Development of the ARMA model for non-stationary time series is the Box-Jenkins model, also known as the ARIMA model, which integrates AR and MA with successive difference/lag operator, ∇^d. Hence, an ARIMA model of order $(p,\ d,\ q)$ is defined

$$\hat{Z}_t = e_t + \sum_{i=1}^{p} \alpha_i Z_{t-i} + \sum_{i=1}^{q} \beta_i e_{t-i} \tag{5.4}$$

where p, q has the previously mentioned definition, and d is the order of nonseasonal successive difference required to make the time series stationary i.e.

- If d=1 then $Z_t = \nabla Y_t = Y_t - Y_{t-1}$
- If d=2 then $Z_t = \nabla^2 Y_t = (Y_t - Y_{t-1}) - (Y_{t-1} - Y_{t-2}) = Y_t - 2Y_{t-1} + Y_{t-2}$
- If d=3 then $Z_t = \nabla^3 Y_t = (Y_t - 2Y_{t-1} + Y_{t-2}) - (Y_{t-1} - 2Y_{t-2} + Y_{t-3}) = Y_t - 3Y_{t-1} + 3Y_{t-2} - Y_{t-3}$
- and so on.

The idea of seasonality using the Fourier coefficient naming Fourier ARIMA model was introduced by [54].

$$Z_t = \delta_0 + \sum_{i=1}^{p} \alpha_l Z_{t-i} + \sum_{j=1}^{q} \beta_J e_{t-j} + \sum_{k=1}^{r} \left[a_k \sin(\omega_k t) + b_k \cos(\omega_k t)\right] Z_{t-m} + e_t \tag{5.5}$$

where, δ_0 is the constant term and ω_k is the periodicity of the data.

We aim to forecast the monthly and synthesized daily data using the forecasting mentioned above techniques and compare the forecast accuracy based on error measures. We use SARIMA and Fourier-ARIMA models to forecast the monthly and synthesized data. The model in each case is chosen based on the lowest value of Akaike's Information Criterion (AIC), Akaike's Information Criterion correction (AICc), and Bayesian Information Criterion (BIC).

5.1 Model Selection Method

Box-Jenkins method is a generalized model selection pathway which works for time series irrespective of its stationarity or seasonality. The method is illustrated in Figure 24.

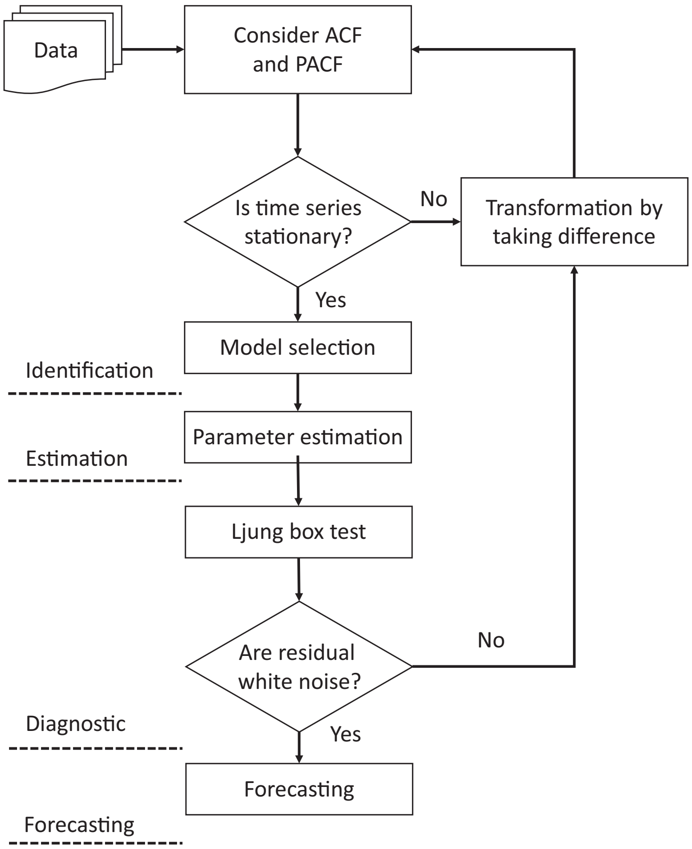

Figure 24: Flow chart of Box-Jenkin's Method.

5.2 Error Measures

The error measures for comparison is Mean Absolute Scaled Error(MASE) which is defined as

$$MASE = \frac{\frac{1}{n}\sum_{i=1}^{n}\left|Y_i - \hat{Y}_i\right|}{\frac{1}{T-m}\sum_{t=m+1}^{T}|Y_t - Y_{t-m}|}$$

We used this metric as it is scale-independent; hence is perfect for comparison. We also could have taken MAPE as a metric, but MAPE is undefined for such cases as the data is populated with zero values. We also use RMSE and MAE to gauge the error in the forecast.

5.3 Forecast on the Aggregated Data

The actual data is monthly Dengue infection data of Bangladesh from 2010 to July 2022. Following Box-Jenkin's method, we firstly check for the stationarity of the data based on the Augmented Dicky Fuller (ADF) test. ADF test returns the value of -4.7906 with p-value = 0.01, which implies that the data is stationary.

We run multiple SARIMA models and calculate their AIC, AICc and BIC and the best model is chosen based on the minimum value of the criterion. We present 5 of the top results in table 6.

Model	AIC	AICc	BIC
SARIMA$(1, 0, 0)(0, 1, 1)_{12}$	2603.57	2603.76	2612.22
SARIMA$(1, 0, 0)(0, 1, 2)_{12}$	2506.58	2506.91	2517.96
SARIMA$(1, 0, 0)(1, 1, 1)_{12}$	2507.12	2507.45	2518.49
SARIMA$(1, 0, 1)(0, 1, 1)_{12}$	2510.07	2510.39	2521.44
SARIMA$(2, 0, 0)(0, 1, 1)_{12}$	2510.13	2510.45	2521.5

Table 6: Selection of Best model based on criteria.

Here, the best model to use is SARIMA $(1, 0, 0)(0, 1, 1)_{12}$. We fit the given model, which gives us the following coefficients:

	ar1	ar2	sma1
	0.6000	-0.0919	-0.8324
S.E.	0.0879	0.0877	0.0948

Table 7: Coefficients of SARIMA $(1, 0, 0)(0, 1, 1)_{12}$ model to fit and forecast actual monthly data of Dengue infection in Bangladesh from 2010 to July, 2022. Here, ar implies autoregressive, ma implies moving average, SMA implies seasonal moving average, and the trailing number enumerates their coefficient ordering. SE implies the standard error of the mean.

To check the goodness of fit of the model, we use the Ljung box test, which returns the p-value $= 0.9998 > 0.05$, i.e. we accept the null hypothesis: *"The model does not show lack ness of fit/ the residuals are not autocorrelated/ the residuals are random white noise."*

Given everything in place, we forecast the infection for the rest of 2023, i.e. from August to December. The forecast is illustrated in the given figure.

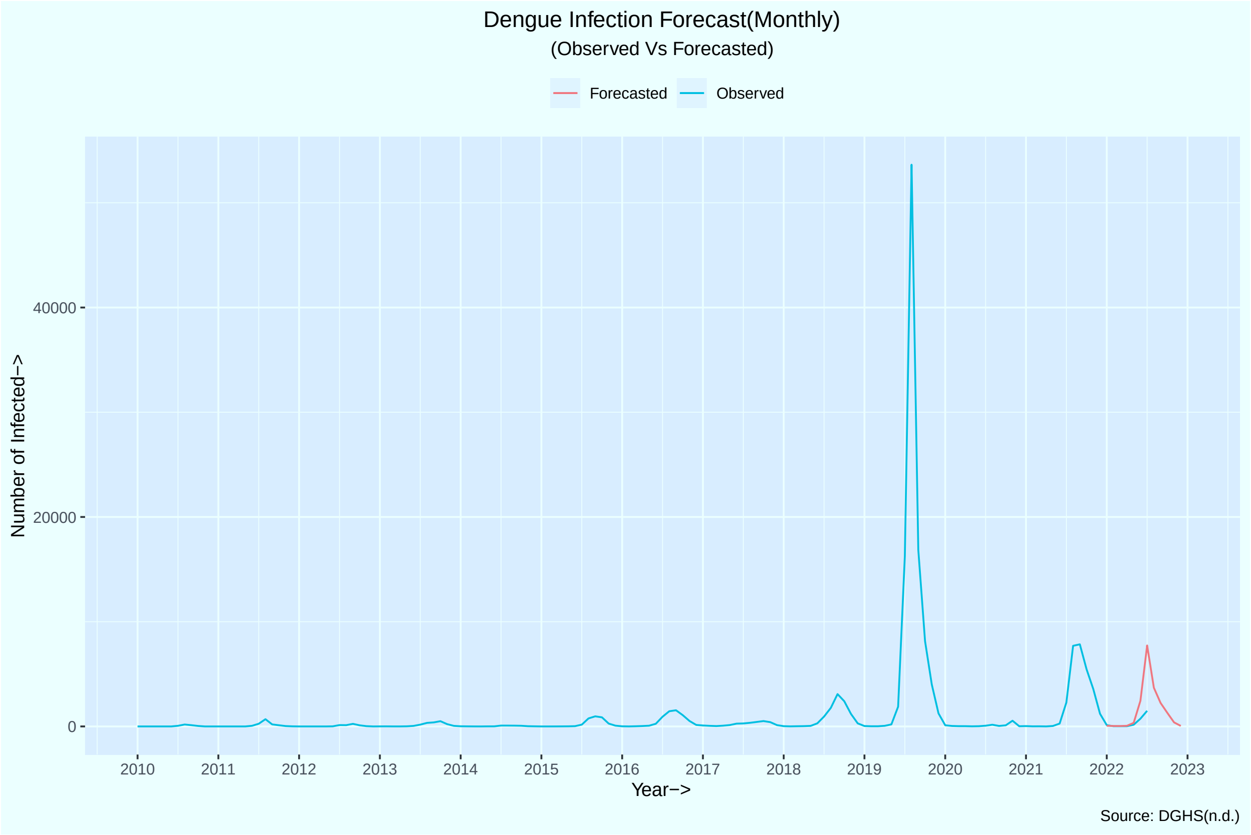

Figure 25: The figure illustrates the forecast generated by SARIMA $(1, 0, 0)(0, 1, 1)_{12}$ from actual aggregated data.

To validate the goodness of the fit, we can analyze the model residual, illustrated in Figure 26. Here, the top graph is the residual with the timeline of the original data. The bottom left graph represents the Autocorrelation Function (ACF) with respect to the lag of the data. Almost all the values are within the significance e level, and the bottom right figure shows the distribution of the model's residuals. It implies that the residuals are distributed generally with zero means.

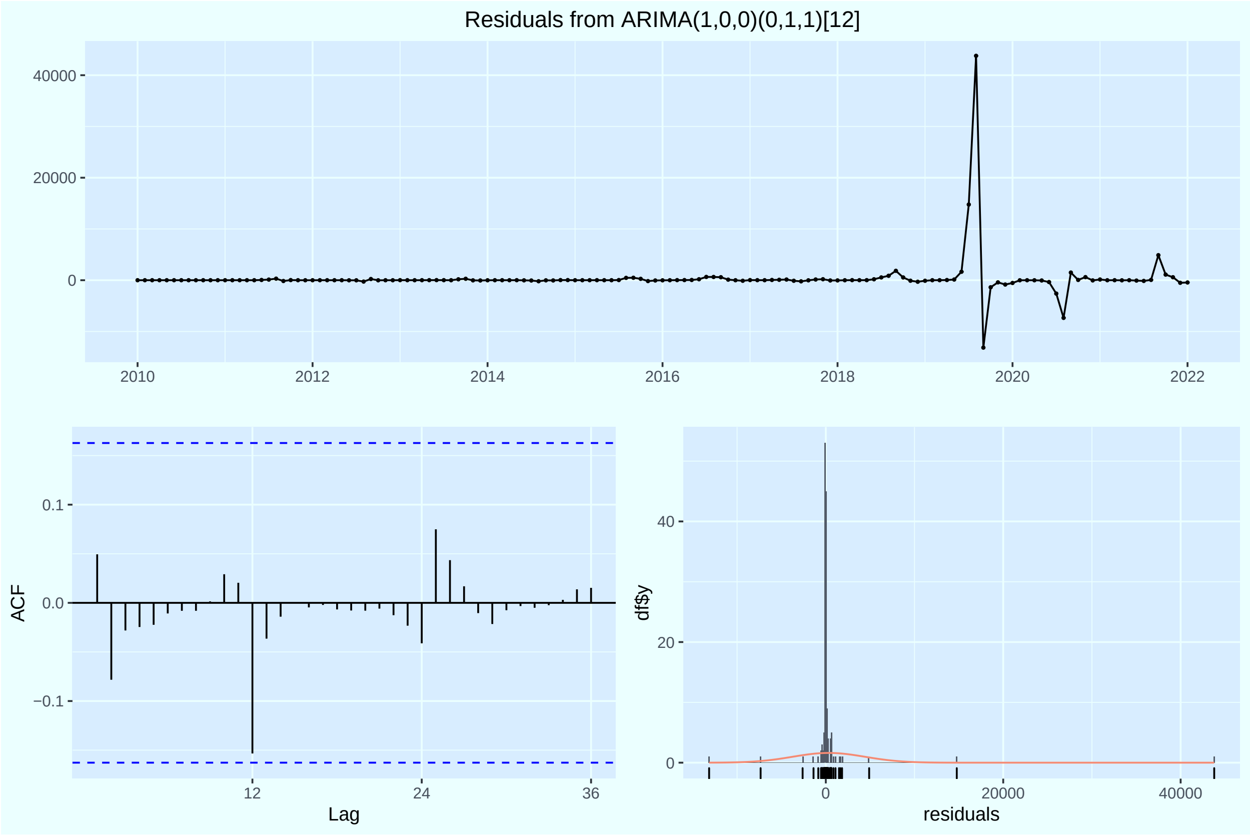

Figure 26: Residual of the SARIMA $(1, 0, 0)(0, 1, 1)_{12}$.

To calculate the accuracy of the given forecast, we calculate the aforementioned error measures.

Data	RMSE	MAE	MASE
Monthly	4092.712	753.6765	0.409654

Table 8: Error measures for the forecast of the SARIMA $(1, 0, 0)(0, 1, 1)_{12}$ of the actual aggregated data.

The error measures are acceptable given the magnitude of the data, but there is room for improvement shall be demonstrated in the following subsection.

5.4 Forecast on the Synthesized Data

The synthesized data is daily Dengue infection data of Bangladesh from 2010 to July 2022. Following Box-Jenkin's method, we firstly check for the stationarity of the data based on the Augmented Dicky Fuller (ADF) test. ADF test returns the value of -6.6531 with p-value = 0.01, which implies that the data is stationary.

We run multiple Fourier ARIMA models and calculate their AIC, AICc and BIC. The best model is chosen based on the minimum value of the criterion. We present 5 of the top results in table 9. Here in each case of Fourier transformation, we used one pair of trigonometric terms where each pair is comprised of a sine and a cosine term as defined in (5.5) and the periodicity of the Fourier term is used to be 365.25. Prior to this we have used box cox transformation of $\lambda = 0.49$

Model	AIC	AICc	BIC
ARIMA(7,0,7)	21711.25	21711.4	21827.03
ARIMA(5,0,0)	21819.04	21819.08	21876.94
ARIMA(3,0,0)	22147.88	22147.9	22192.91
ARIMA(2,0,0)	22527.02	22527.04	22565.61
ARIMA(1,0,0)	23476.24	23476.25	23508.4
ARIMA(0,0,0)	33245.98	33271.71	33271.71

Table 9: Selection of Best model based on criteria.

Here, the best model to use is ARIMA(7,0,7). We fit the given model, which gives us the following coefficients:

	ar1	ar2	ar3	ar4	ar5	ar6	ar7	ma1
	-0.5273	0.3109	1.2946	1.0562	0.2775	-0.6222	-0.7940	0.8055
S.E.	0.0513	0.0310	0.0419	0.0755	0.0323	0.0353	0.0488	0.0471
	ma2	ma3	ma4	ma5	ma6	ma7	intercept	s1-365
	0.0718	-1.0032	-1.1256	-0.5327	0.3051	0.6454	3.3498	6.6197
S.E.	0.0340	0.0365	0.0602	0.0321	0.0356	0.0303	1.4789	1.6859
	c1-365							
	-0.7430							
S.E.	1.6857							

Table 10: Coefficients of ARIMA(7,0,7) model to fit and forecast actual monthly data of Dengue infection in Bangladesh from 2010 to July 2022. Here, ar implies auto-regressive, ma implies the moving average, s and c represent the coefficient of the sine and cosine of Fourier term, intercept implies the constant term, and the trailing number enumerates their coefficient ordering. SE implies the standard error of the mean.

To check the goodness of fit of the model, we use the Ljung box test, which returns the p-value $= 0.07749 > 0.05$, i.e. we accept the null hypothesis: *"The model does not show lack ness of fit/ the residuals are not autocorrelated/ the residuals are random white noise"*.

Given everything in place, we forecast the infection for the rest of 2023, i.e. from August to December. The forecast is illustrated in the given figure.

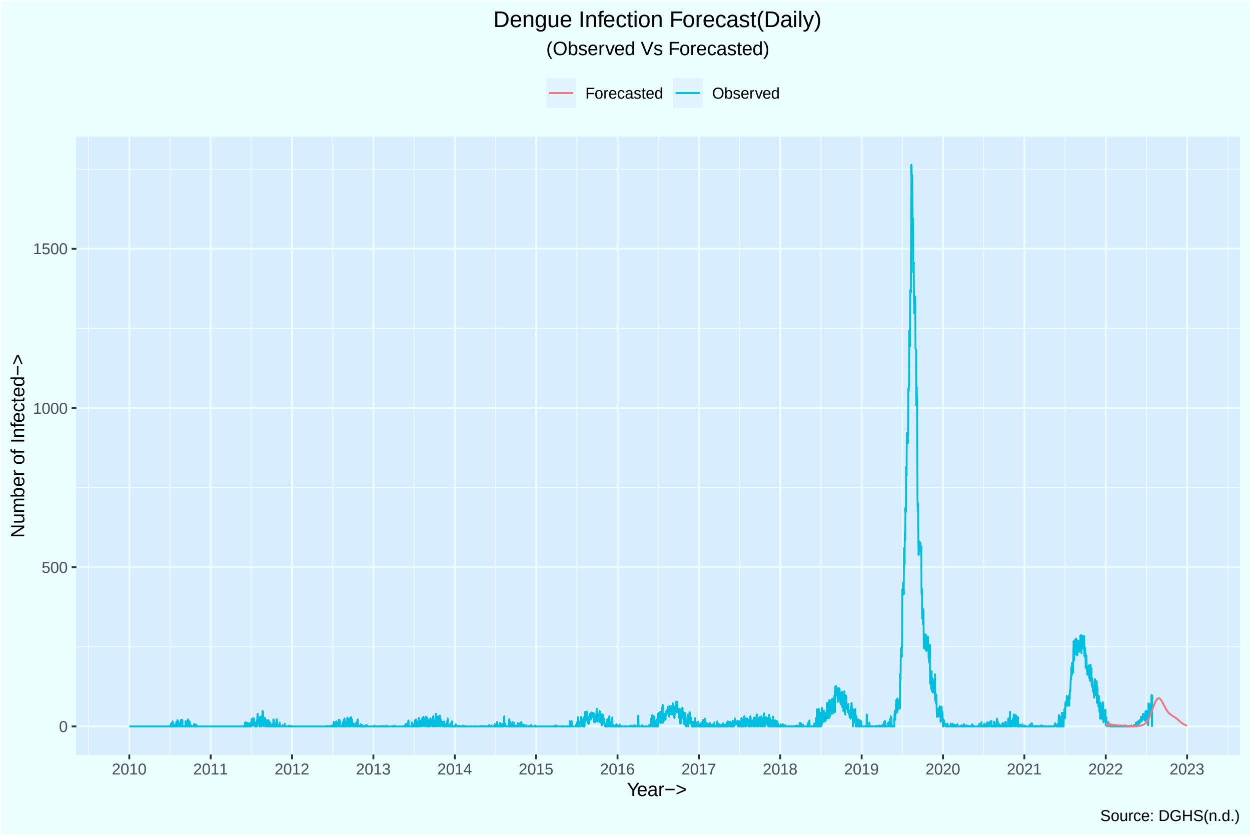

Figure 27: The figure illustrates the forecast generated by ARIMA(7,0,7) from actual aggregated data.

To validate the goodness of the fit, we can analyze the model residual, illustrated in Figure 28. Here, the top graph is that of the residual with the timeline of the original data. The bottom left graph represents the Autocorrelation Function (ACF) with respect to the lag of the data. Almost all the values are within the significance e level, and the bottom right figure shows the distribution of the model's residuals. It implies that the residuals are distributed normally with zero mean.

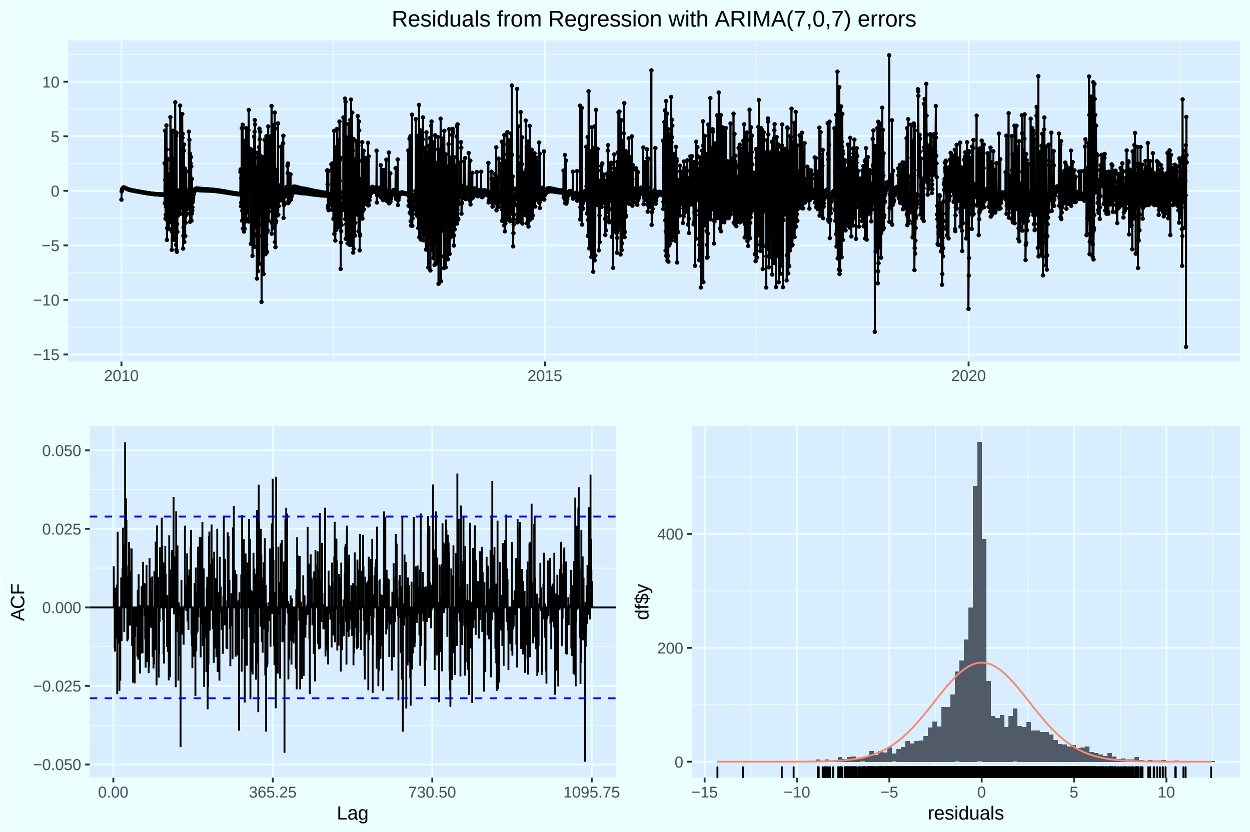

Figure 28: Residual of the ARIMA (7, 0, 7).

To calculate the accuracy of the given forecast, we calculate the aforementioned error measures.

Data	RMSE	MAE	MASE
Daily	18.71255	6.593062	0.1115845

Table 11: Error measures for the forecast of the ARIMA (7, 0, 7) of the synthetic daily data.

The error measures are acceptable, given the magnitude of the data. In comparison to the error measures of the actual data illustrated in table 8, we can see improvement in the table 11. Comparing the MASE term of the two tables shows about four times improvement in the forecast accuracy using the synthetic data over actual data.

6 Conclusion

Downscaling algorithm has been predominantly used in geology to facilitate outputs of the prevalent models in the field. Very few applications have been made in epidemiology, and most of the application is spatial downscaling. This paper contributes by proposing a parametric, probabilistic one-dimensional downscaling algorithm using aggregated data in the field of epidemiology that facilitates existing forecasting tools box to generate better forecasts than the aggregated data. The SBD algorithm is by no means by construction applies to only epidemiological data but is tuned to work with non-negative, integer data. Deduction of particular conditioning on the initial data synthesizer can generalize the model to practical and real data. This opens up a horizon for

applying the SBD algorithm on the subject of temporal downscaling. Other than this, the SBD algorithm can be a potent deconvolution algorithm to recover Gaussian blurring and generate high-resolution images keeping the mean pixel value of the image unchanged. There are still avenues to be explored using this algorithm. Further work is needed to show how the SBD algorithm's output affects the outcome of predictive machine learning models like Long Short Term Memory (LSTM), and prophet.

Acknowledgments

The research by M. Kamrujjaman was partially supported by the University Grants Commission (UGC), and the University of Dhaka, Bangladesh.

Declaration of generative AI and AI-assisted technologies in the writing process

During the preparation of this work the author(s) never used any tools/service of AI and AI-assisted technologies.

Conflict of interest

The authors declare no conflict of interest.

Ethical approval

No consent is required to publish this manuscript.

Author contributions

Conceptualization, MK and MAM; methodology, MAM and MK; software, MAM and MK; validation, MK; formal analysis, MAM; investigation, MK; data curation, MAM; original draft preparation, MAM; review and editing, MK; supervision, MK. Both authors have read and agreed to the published version of the manuscript.

References

[1] Jian Peng, Alexander Loew, Olivier Merlin, and Niko EC Verhoest. A review of spatial downscaling of satellite remotely sensed soil moisture. *Reviews of Geophysics*, 55(2):341–366, 2017.

[2] J Ribalaygua, L Torres, J Pórtoles, R Monjo, E Gaitán, and MR Pino. Description and validation of a two-step analogue/regression downscaling method. *Theoretical and Applied Climatology*, 114(1):253–269, 2013.

[3] Seon-Ho Kim, Jeong-Bae Kim, and Deg-Hyo Bae. Optimizing parameters for the downscaling of daily precipitation in normal and drought periods in south korea. *Water*, 14(7):1108, 2022.

[4] Deg-Hyo Bae, Toshio Koike, Jehangir Ashraf Awan, Moon-Hwan Lee, and Kyung-Hwan Sohn. Climate change impact assessment on water resources and susceptible zones identification in the asian monsoon region. *Water Resources Management*, 29(14):5377–5393, 2015.

[5] Moon-Hwan Lee, Eun-Soon Im, and Deg-Hyo Bae. Impact of the spatial variability of daily precipitation on hydrological projections: A comparison of gcm-and rcm-driven cases in the han river basin, korea. *Hydrological Processes*, 33(16):2240–2257, 2019.

[6] Jeong-Bae Kim, Eun-Soon Im, and Deg-Hyo Bae. Intensified hydroclimatic regime in korean basins under 1.5 and 2° c global warming. *International Journal of climatology*, 40(4):1965–1978, 2020.

[7] Subhrendu Gangopadhyay, Martyn Clark, and Balaji Rajagopalan. Statistical downscaling using k-nearest neighbors. *Water Resources Research*, 41(2), 2005.

[8] Hayley J Fowler, Stephen Blenkinsop, and Claudia Tebaldi. Linking climate change modelling to impacts studies: recent advances in downscaling techniques for hydrological modelling. *International Journal of Climatology: A Journal of the Royal Meteorological Society*, 27(12):1547–1578, 2007.

[9] Taesam Lee and Changsam Jeong. Nonparametric statistical temporal downscaling of daily precipitation to hourly precipitation and implications for climate change scenarios. *Journal of Hydrology*, 510:182–196, 2014.

[10] Grant Buster, Michael Rossol, Galen Maclaurin, Yu Xie, and Manajit Sengupta. A physical downscaling algorithm for the generation of high-resolution spatiotemporal solar irradiance data. *Solar Energy*, 216:508–517, 2021.

[11] Jiaming Liu, Di Yuan, Liping Zhang, Xia Zou, and Xingyuan Song. Comparison of three statistical downscaling methods and ensemble downscaling method based on bayesian model averaging in upper hanjiang river basin, china. *Advances in Meteorology*, 2016, 2016.

[12] Liao Yaoming, Zhang Qiang, and Chen Deliang. Stochastic modeling of daily precipitation in china. *Journal of Geographical Sciences*, 14(4):417–426, 2004.

[13] Yaoming Liao. Change of parameters of bcc/rcg-wg for daily non-precipitation variables in china: 1951–1978 and 1979–2007. *Journal of Geographical Sciences*, 23(4):579–594, 2013.

[14] Yonas B Dibike and Paulin Coulibaly. Hydrologic impact of climate change in the saguenay watershed: comparison of downscaling methods and hydrologic models. *Journal of hydrology*, 307(1-4):145–163, 2005.

[15] Mohammad Sajjad Khan, Paulin Coulibaly, and Yonas Dibike. Uncertainty analysis of statistical downscaling methods. *Journal of Hydrology*, 319(1-4):357–382, 2006.

[16] Robert L Wilby, Christian W Dawson, and Elaine M Barrow. Sdsm—a decision support tool for the assessment of regional climate change impacts. *Environmental Modelling & Software*, 17(2):145–157, 2002.

[17] Colin Harpham and Robert L Wilby. Multi-site downscaling of heavy daily precipitation occurrence and amounts. *Journal of Hydrology*, 312(1-4):235–255, 2005.

[18] Robert L Wilby and Michael D Dettinger. Streamflow changes in the sierra nevada, california, simulated using a statistically downscaled general circulation model scenario of climate change. In *Linking climate change to land surface change*, pages 99–121. Springer, 2000.

[19] Fredrik Wetterhall, Sven Halldin, and C-Y Xu. Seasonality properties of four statistical-downscaling methods in central sweden. *Theoretical and Applied Climatology*, 87(1):123–137, 2007.

[20] Adrian E Raftery and Yingye Zheng. Discussion: Performance of bayesian model averaging. *Journal of the American Statistical Association*, 98(464):931–938, 2003.

[21] Shivam Tripathi, VV Srinivas, and Ravi S Nanjundiah. Downscaling of precipitation for climate change scenarios: a support vector machine approach. *Journal of hydrology*, 330(3-4):621–640, 2006.

[22] Xinying Yu and Shie-Yui Liong. Forecasting of hydrologic time series with ridge regression in feature space. *Journal of Hydrology*, 332(3-4):290–302, 2007.

[23] Subimal Ghosh and Pradeep P Mujumdar. Statistical downscaling of gcm simulations to streamflow using relevance vector machine. *Advances in water resources*, 31(1):132–146, 2008.

[24] Timothy C Matisziw, Tony H Grubesic, and Hu Wei. Downscaling spatial structure for the analysis of epidemiological data. *Computers, Environment and Urban Systems*, 32(1):81–93, 2008.

[25] Md Shahriar Mahmud, Md Kamrujjaman, Md Mashih Ibn Yasin Adan, Md Alamgir Hossain, Md Mizanur Rahman, Md Shahidul Islam, Muhammad Mohebujjaman, and Md Mamun Molla. Vaccine efficacy and sars-cov-2 control in california and us during the session 2020–2026: A modeling study. *Infectious Disease Modelling*, 7(1):62–81, 2022.

[26] DGHS. Denv press relseases, 2022.

[27] IEDCR. Dengue surveillence report, September 2021.

[28] WHO. Covid-19 dashboard, 2022.

[29] Rob J Hyndman and Anne B Koehler. Another look at measures of forecast accuracy. *International journal of forecasting*, 22(4):679–688, 2006.

[30] Robert Gilmore Pontius, Olufunmilayo Thontteh, and Hao Chen. Components of information for multiple resolution comparison between maps that share a real variable. *Environmental and ecological statistics*, 15(2):111–142, 2008.

[31] Cort J Willmott and Kenji Matsuura. On the use of dimensioned measures of error to evaluate the performance of spatial interpolators. *International Journal of Geographical Information Science*, 20(1):89–102, 2006.

[32] Rob J Hyndman and George Athanasopoulos. *Forecasting: principles and practice.* OTexts, 2018.

[33] Md Shahidul Islam, Jannatun Irana Ira, KM Ariful Kabir, and Md Kamrujjaman. Effect of lockdown and isolation to suppress the covid-19 in bangladesh: an epidemic compartments model. *J Appl Math Comput*, 4(3):83–93, 2020.

[34] Md Kamrujjaman, Md Shahriar Mahmud, and Md Shafiqul Islam. Coronavirus outbreak and the mathematical growth map of covid-19. *Annual Research & Review in Biology*, pages 72–78, 2020.

[35] Nor Azura Husin, Naomie Salim, et al. Modeling of dengue outbreak prediction in malaysia: a comparison of neural network and nonlinear regression model. In *2008 International Symposium on Information Technology*, volume 3, pages 1–4. IEEE, 2008.

[36] Li Ping Wong, Sharina Mahavera Mohamad Shakir, Narges Atefi, and Sazaly AbuBakar. Factors affecting dengue prevention practices: nationwide survey of the malaysian public. *PloS one*, 10(4):e0122890, 2015.

[37] Liang Lu, Hualiang Lin, Linwei Tian, Weizhong Yang, Jimin Sun, and Qiyong Liu. Time series analysis of dengue fever and weather in guangzhou, china. *BMC Public Health*, 9(1):1–5, 2009.

[38] Sunil Bhatnagar, Vivek Lal, Shiv D Gupta, Om P Gupta, et al. Forecasting incidence of dengue in rajasthan, using time series analyses. *Indian journal of public health*, 56(4):281, 2012.

[39] S Wongkoon, M Pollar, M Jaroensutasinee, and K Jaroensutasinee. Predicting dhf incidence in northern thailand using time series analysis technique. *International Journal of Medical and Health Sciences*, 1(8):484–488, 2007.

[40] Myriam Gharbi, Philippe Quenel, Joël Gustave, Sylvie Cassadou, Guy La Ruche, Laurent Girdary, and Laurence Marrama. Time series analysis of dengue incidence in guadeloupe, french west indies: forecasting models using climate variables as predictors. *BMC infectious diseases*, 11(1):1–13, 2011.

[41] Claudia Torres, Samier Barguil, Miguel Melgarejo, and Andrés Olarte. Fuzzy model identification of dengue epidemic in colombia based on multiresolution analysis. *Artificial intelligence in medicine*, 60(1):41–51, 2014.

[42] Paula M Luz, Beatriz VM Mendes, Claudia T Codeço, Claudio J Struchiner, Alison P Galvani, et al. Time series analysis of dengue incidence in rio de janeiro, brazil. 2008.

[43] Wenbiao Hu, Archie Clements, Gail Williams, and Shilu Tong. Dengue fever and el nino/southern oscillation in queensland, australia: a time series predictive model. *Occupational and environmental medicine*, 67(5):307–311, 2010.

[44] Faruq Abdulla and Md Moyazzem Hossain. Forecasting of wheat production in kushtia district & bangladesh by arima model: An application of box-jenkin's method. *Journal of Statistics Applications & Probability*, 4(3):465, 2015.

[45] Md Moyazzem Hossain and Faruq Abdulla. Forecasting potato production in bangladesh by arima model. *Journal of Advanced Statistics*, 1(4):191–198, 2016.

[46] MM Hossain and F Abdulla. Jute production in bangladesh: a time series analysis. *Journal of Mathematics and Statistics*, 11(3):93–98, 2015.

[47] Md Hossain, Faruq Abdulla, et al. Forecasting the tea production of bangladesh: Application of arima model. 2015.

[48] M Hossian and F Abdulla. A time series analysis for the pineapple production in bangladesh. *Jahangirnagar University Journal of Science*, 38(2):49–59, 2015.

[49] Arul Earnest, Say Beng Tan, Annelies Wilder-Smith, and David Machin. Comparing statistical models to predict dengue fever notifications. 2012:1–6, 2012.

[50] Matthew D Eastin, Eric Delmelle, Irene Casas, Joshua Wexler, and Cameron Self. Intra-and interseasonal autoregressive prediction of dengue outbreaks using local weather and regional climate for a tropical environment in colombia. *The American journal of tropical medicine and hygiene*, 91(3):598, 2014.

[51] Pei-Chih Wu, How-Ran Guo, Shih-Chun Lung, Chuan-Yao Lin, and Huey-Jen Su. Weather as an effective predictor for occurrence of dengue fever in taiwan. *Acta tropica*, 103(1):50–57, 2007.

[52] Edson Zangiacomi Martinez, Elisângela Aparecida Soares da Silva, and Amaury Lelis Dal Fabbro. A sarima forecasting model to predict the number of cases of dengue in campinas, state of são paulo, brazil. *Revista da Sociedade Brasileira de Medicina Tropical*, 44:436–440, 2011.

[53] Jason Brownlee. *Introduction to time series forecasting with python: how to prepare data and develop models to predict the future*. Machine Learning Mastery, 2017.

[54] Dilip Nachane and Jose G Clavel. Forecasting interest rates: a comparative assessment of some second-generation nonlinear models. *Journal of Applied Statistics*, 35(5):493–514, 2008.

[55] Iberedem Iwok and G Udoh. A comparative study between the arima-fourier model and the wavelet model 1. *AMERICAN JOURNAL OF SCIENTIFIC AND INDUSTRIAL RESEARCH*, 7:137–144, 12 2016.

Click here to access/download
Preprint PDF
Preprint_arXiv_2304.12305.pdf
