# Supplementary material for: Downscaling epidemiological time series data for improving forecasting accuracy: An algorithmic approach
Source: PLoS One. 2023 Dec 14;18(12):e0295803. doi: 10.1371/journal.pone.0295803 (PMC10721108; doi:10.1371/journal.pone.0295803)
Supplement: Attachment — Submitted filename: Response to Reviewers_R1_PONE-D-23-27740.pdf [file pone.0295803.s005.pdf]

Md. Kamrujjaman, Ph.D.
Professor
Department of Mathematics
University of Dhaka
Dhaka-1000, Bangladesh
E-mail: kamrujjaman@gmail.com
kamrujjaman@du.ac.bd

November 13, 2023

Editor-in-Chief/Academic Editor
PLOS ONE

Dear Professor Dr. Salim Heddam

Please find the revised version of the manuscript (Manuscript ID: PONE-D-23-27740) entitled: **"Downscaling Epidemiological Time Series Data for Improving Forecasting Accuracy: An Algorithmic Approach"**, and consider it for publication in the journal **PLOS ONE**.

We have revised the manuscript according to the Editors/Reviewers comments and suggestions.

Authors' Note: The major corrections and new addendum are highlighted in magenta color texts for Reviewer 1 and cyan color texts for Reviewer 2 in the revised manuscript. Following journal template additional corrections are highlighted in **a different color**.

Legend (texts colors for revision and corrections)

- Reviewer 1
- Reviewer 2
- Authors'

Journal Queries

Queries and Corresponding Response:

1. Please remove your figures/ from within your manuscript file, leaving only the individual TIFF/EPS image files. These will be automatically included in the reviewer's PDF.
   Ans: Done in the revised version.

2. Please ensure that you refer to Tables 2,3,5 and 7 in your text as, if accepted, production will need this reference to link the reader to the Table.
   Ans: Fully agree and the tables are uploaded separately.

3. As you may already be aware, PLOS journals require authors to make all data underlying the findings described in their manuscript fully available without restriction at the time of publication (https://journals.plos.org/plosone/s/data-availability). PLOS only allows data to be available upon request if there are legal or ethical restrictions on sharing data publicly. For more information on unacceptable data access restrictions, please see http://journals.plos.org/plosone/s/data-availability#loc-unacceptable-data-access-restrictions.
   1] (For the dengue data) Before we proceed with your manuscript, please address the following prompts:
   a) If there are ethical or legal restrictions on sharing the data set, please explain them in detail (e.g., data contain potentially identifying or sensitive patient information, data are owned by a third-party organization, etc.) and who has imposed them (e.g., a Research Ethics Committee or Institutional Review Board, etc.). Please also provide non-author contact information (email address) for a data access committee, ethics committee, or other institutional body to which data requests may be sent.
   Ans: Fortunately there are no ethical or legal restrictions on sharing the data set, as the data has been made public by the government recently for research purposes by the dashboard on the link (https://lookerstudio.google.com/embed/u/0/reporting/6b10cb45-90c1-4895-be2e-6a438aea3d0f/page/vDc8C)
   b) If there are no restrictions, please upload the minimal anonymised data set necessary to replicate your study findings to a stable, public repository and provide us with the relevant URLs, DOIs, or accession numbers. For a list of recommended repositories, please see https://journals.plos.org/plosone/s/recommended-repositories. You also have the option of uploading the data as Supporting Information files, but we would recommend depositing data directly to a data repository if possible.
   Ans: The minimal anonymised data set necessary to replicate our study findings has been uploaded in the Supporting Information files.
   2] (For the COVID-19 Data) Please also note that the data we ask authors to share is not necessarily the full and/or raw data set collected in the course of their research, but rather the "minimal data set", which we define as consisting of the data set used to reach the conclusions drawn in the manuscript with related metadata and methods, and any additional data required to replicate the reported study findings in their entirety. Depending on the nature of your study, this can include any or all of the following:
   i) The values behind the means, standard deviations and other measures reported;
   ii) The values used to build graphs;
   iii) The points extracted from images for analysis.
   Can you make a de-identified, anonymized, or aggregated data set publicly available in a repository or, if that is not possible, in your manuscript's Supporting Information

files? For a list of recommended repositories, some of which are able to hold sensitive data, see here: https://journals.plos.org/plosone/s/recommended-repositories
Ans: The data set necessary to replicate our study findings has been uploaded in the Supporting Information files.

Journal Requirements/Academic Editor

Comments and Corresponding Answers:

1. Please ensure that your manuscript meets PLOS ONE's style requirements, including those for file naming. The PLOS ONE style templates can be found at https://journals.plos.org/plosone/s/file?id=wjVg/PLOSOne_formatting_sample_main_body.pdf and https://journals.plos.org/plosone/s/file?id=ba62/PLOSOne_formatting_sample_title_auths_affiliations.pdf
Ans: The revised manuscript is prepared using the PLOS ONE journal template. We have shared the latex source file and all figures to compile the paper.

2. Note from Emily Chenette, Editor in Chief of PLOS ONE, and Iain Hrynaszkiewicz, Director of Open Research Solutions at PLOS: Did you know that depositing data in a repository is associated with up to a 25% citation advantage (https://doi.org/10.1371/journal.pone.0230416)? If you've not already done so, consider depositing your raw data in a repository to ensure your work is read, appreciated and cited by the largest possible audience.
Ans: We have uploaded the csv file as Supplementary files.

3. Please note that PLOS ONE has specific guidelines on code sharing for submissions in which author-generated code underpins the findings in the manuscript. In these cases, all author-generated code must be made available without restrictions upon publication of the work. Please review our guidelines at https://journals.plos.org/plosone/s/materials-and-software-sharing#loc-sharing-code and ensure that your code is shared in a way that follows best practice and facilitates reproducibility and reuse.
Ans: The programming code is shared directly for future research and upgrading by other researchers.

4. In your Data Availability statement, you have not specified where the minimal data set underlying the results described in your manuscript can be found. PLOS defines a study's minimal data set as the underlying data used to reach the conclusions drawn in the manuscript and any additional data required to replicate the reported study findings in their entirety. All PLOS journals require that the minimal data set be made fully available. For more information about our data policy, please see http://journals.plos.org/plosone/s/data-availability.
Ans: The minimal data sets are uploaded as .csv file. Both dengue and Covid-19 data are shared which we have studied in this research.

Reviewer 1

1. Add a projection value in the end of the abstract.
   Ans: Thank you for your suggestion. The paper aims to deal with error reduction for classical statistical model using synthetic data with respect to actual data. Projection value of forecasting is added and the abstract is revised. Please check the updated version and at a glance –
   $\cdots$ **The paper presents two epidemiological time series case studies of Bangladesh (Dengue, Covid-19) to showcase the workflow of the algorithm. The case studies illustrate that the synthesized data agrees with the original data regarding its statistical properties, trend, seasonality, and residuals. In the case of forecasting performance, using the last 12 years data of Dengue infection data in Bangladesh, we were able to decrease error terms up to 72.76% using synthetic data over actual aggregated data.**
2. Add objectives of the paper pointwise before organization of the paper.
   Ans: Noted, included and revised. Please check page 2 in the updated version. Immediately–
   $\cdots$ **The paper aims to achieve the following:**
   - **To propose a novel algorithm named Stochastic Bayesian Downscaling (SBD) algorithm based on the Bayesian approach that can regenerate downscaled temporal time series of varying time lengths from aggregated data preserving most of the statistical characteristics and the aggregated sum of the original data.**
   - **To present two downscaling case studies of epidemiological time series data (namely Dengue and COVID-19 data of Bangladesh) to showcase the workflow and efficacy of the algorithm.**
   - **To present a comparison between the forecasting performance of aggregated data and algorithm generated synthetic data to showcase the improvement achieved (for synthetic data over aggregated data) in terms of scale independent error.**
3. In the data section, add link to the data source.
   Ans: The link to the data sources has been mentioned in the reference section. Right away-
   For dengue: 1. DGHS DENV Press Relseases (2022),
   https://dashboard.dghs.gov.bd/webportal/pages/heoc dengue.php
   2. IEDCR Dengue Surveillence Report, https://iedcr.gov.bd/surveillances/

   Covid data is publicly available.

   Also we have uploaded the csv file as supplementary files.
4. In the preprocessing and result section, mention which statistical software used for the

study.
Ans: The statistical software used for this paper is R which has been revised in the mentioned section. See page-8 in the revised manuscript.

5. Explain the table 3 properly.
Ans: Thank you for your suggestion. It's revised and see page-9, instantly check the following–
··· **This table 3 illustrates the comparison of the basic statistical measures of the synthesized data with respect to the actual data. The second and third column represent the statistical measures observed for the actual data and algorithm generated data respectively. The synthetic data was able to replicate the mean of the actual data exactly with out being provided with it. The rest of the statistics are close enough approximation except that of the maximum value. A smart way to achieve this is can be an open avenue for research.**

6. In the conclusion section, highlight the key finding of the study.
Ans: Revised on page-17 and partially presented here-
··· **findings show that using synthetic data over actual aggregated data, we were able to reduce the scale-free error measure by 72.76%.**

The SBD algorithm presented in this paper is designed to handle integer data by imposing certain restrictions but can be generalized to handle real numbers upon relaxing such restrictions. Hence, exploring diverse use cases in public health, epidemiology, economics, and finance can be a future direction of research. In this paper, we have only studied how statistical forecasting models respond to synthetic data compared to actual data. Repeating similar studies for the predictive class of machine learning models like Long Short Term Memory (LSTM), XGboost, etc is a further scope of research.

The downscaling algorithm has been predominantly used in geology to facilitate outputs of the prevalent models in the field. Very few applications have been made in epidemiology, and most of the application is spatial downscaling. This paper contributes to the current body of knowledge by proposing a parametric, probabilistic one-dimensional downscaling algorithm using aggregated data in the field of epidemiology that facilitates existing classical statistical forecasting tools box to generate better forecasts than the aggregated data. As we know, forecasting models like ARIMA and SARIMA are sensitive to data discontinuity and outliers, hence, the SBD algorithm can be implemented as pre model cleaning step to curate better results on a finer scale. As the SBD algorithm can increase *data volume to a significant scale (e.g. downscaling monthly data to daily data can increase the number of data points to 30 times on average) while preserving*

***key statistics and properties of the data*, hence such downscaled data can open the avenue for exploration using state of the art neural network model which often requires large volume of data to generate fruitful outcome.**

Reviewer 2

1. The authors should explain the proposed technique in detail.
   Ans: A 5 page detailed explanation of algorithm has been included in the section titled "methodology" (page 3-7). At a glance, partially the revised part is–
   ··· **The algorithm works in three unique phases. The first phase (Initial Data Generator) generates a initial approximation based on the prior distribution, the second phase (Overthrow Correction) removes any abrupt fluctuation introduced during the de-aggregation in the first step, and finally the final step (Volume Correction) rectifies the any displacement of data point volume over the aggregation unit in the second step thus aggregation of this downscaled data agrees with the initial data.**

   ··· **The overthrow correction part takes a tolerance, δ, iteration limit, n, and a radius of an open interval, r. The step initially determines overthrow using tolerance between two neighboring points, i.e., if $y_i - y_{i-1} > \delta$ or $y_i - y_{i+1} > \delta$ then y_i is an overthrow. After identifying an overthrow, we consider an open interval of radius r around the overthrow point and execute the distribution generator on that open interval. This redistributes the sample within the open interval diminishing the overthrow to some extent. This process is iterated n times over the entire time series to ensure satisfactory results. The strength of the overthrow correction step can be dictated by the two parameters δ and n. The strength of the overthrow correction is directly proportional to n and is inversely proportional to δ. Selecting the correct parameter value can ensure a good approximation of the real-life scenario.**

2. Revise abstract and conclusion with specific objectives.
   Ans: Thank you indeed for suggesting this point and we have revised the abstract. Straightaway-
   ··· **and discontinuity are common occurrences in the healthcare and epidemiological dataset and often is needed to form an educative decision and forecast the upcoming scenario. Often to avoid these problems, these data are processed as monthly/yearly aggregate where the prevalent forecasting tools like Autoregressive Integrated Moving Average (ARIMA), Seasonal Autoregressive Integrated Moving Average (SARIMA), and TBATS often fail to provide satisfactory results. Artificial data synthesis methods have been proven to be a powerful tool for tackling these challenges. The paper aims to propose a novel algorithm named Stochastic Bayesian Downscaling (SBD) algorithm based on the Bayesian approach that can regenerate**

downscaled time series of varying time lengths from aggregated data, preserving most of the statistical characteristics and the aggregated sum of the original data. The paper presents two epidemiological time series case studies of Bangladesh (Dengue, Covid-19) to showcase the workflow of the algorithm. The case studies illustrate that the synthesized data agrees with the original data regarding its statistical properties, trend, seasonality, and residuals. In the case of forecasting performance, using the last 12 years data of Dengue infection data in Bangladesh, we were able to decrease error terms up to 72.76% using synthetic data over actual aggregated data.

Similarly, the specific objectives are (see page 2 in the updated version)–

··· **The paper aims to achieve the following:**

- **To propose a novel algorithm named Stochastic Bayesian Downscaling (SBD) algorithm based on the Bayesian approach that can regenerate downscaled temporal time series of varying time lengths from aggregated data preserving most of the statistical characteristics and the aggregated sum of the original data.**
- **To present two downscaling case studies of epidemiological time series data (namely Dengue and COVID-19 data of Bangladesh) to showcase the workflow and efficacy of the algorithm.**
- **To present a comparison between the forecasting performance of aggregated data and algorithm generated synthetic data to showcase the improvement achieved (for synthetic data over aggregated data) in terms of scale independent error.**

Finally the conclusion is revised focusing major findings, please check page-17, Instantaneously–

··· **using synthetic data over actual aggregated data, we were able to reduce the scale-free error measure by 72.76%. The SBD algorithm presented in this paper is designed to handle integer data by imposing certain restrictions but can be generalized to handle real numbers upon relaxing such restrictions. Hence, exploring diverse use cases in public health, epidemiology, economics, and finance can be a future direction of research. In this paper, we have only studied how statistical forecasting models respond to synthetic data compared to actual data. Repeating similar studies for the predictive class of machine learning models like Long Short Term Memory (LSTM), XGboost, etc is a further scope of research.**

The downscaling algorithm has been predominantly used in geology to facilitate outputs of the prevalent models in the field. Very few applications have been made in epidemiology, and most of the application is spatial downscaling. This paper contributes to the current body of knowledge by proposing a parametric, probabilistic one-dimensional downscaling al-

gorithm using aggregated data in the field of epidemiology that facilitates existing classical statistical forecasting tools box to generate better forecasts than the aggregated data. As we know, forecasting models like ARIMA and SARIMA are sensitive to data discontinuity and outliers, hence, the SBD algorithm can be implemented as pre model cleaning step to curate better results on a finer scale. As the SBD algorithm can increase *data volume to a significant scale (e.g. downscaling monthly data to daily data can increase the number of data points to 30 times on average) while preserving key statistics and properties of the data*, hence such downscaled data can open the avenue for exploration using state of the art neural network model which often requires large volume of data to generate fruitful outcome.

3. Precise description of the statistical model and descriptive statistics.
Ans: We thank the Reviewer for this comment. The described review has been incorporated in page 7 subsection titled "Error Measures for Benchmark" and subsection titled "Improvements in Forecasting Accuracy" in page 13. Right off the bat–
··· **To compare the result with the real world data we shall use two error terms that describes the overall error of the approximation. These are as follows:**

- **Root Mean Square Error:**

$$\textbf{RMSE}=\sqrt{\frac{\sum_{i=1}^{N}(x_i-\hat{x}_i)}{N}}$$

- **Mass Absolute Error:**

$$\textbf{MAE}=\frac{\sum_{i=1}^{N}|x_i-\hat{x}_i|}{N}$$

where, x_i is the actual data and $\hat{x}_i$ is the predicted data.

Gradually, the statistics review has been incorporated in page subsection titled "Improvements in Forecasting Accuracy" in page 13. On the spot–
··· **The idea of seasonality using the Fourier coefficient naming Fourier ARIMA model was introduced by [51].**

$$Z_t=\delta_0+\sum_{i=1}^{p}\alpha_l Z_{t-i}+\sum_{j=1}^{q}\beta_J e_{t-j}+\sum_{k=1}^{r}\left[a_k\sin\left(\omega_k t\right)+b_k\cos\left(\omega_k t\right)\right]Z_{t-m}+e_t \qquad (1)$$

where, δ_0 is the constant term and ω_k is the periodicity of the data.

We aim to forecast the monthly and synthesized daily data using the forecasting mentioned above techniques and compare the forecast accuracy based on error measures. We use SARIMA and Fourier-ARIMA models

to forecast the monthly and synthesized data. The model in each case is chosen based on the lowest value of Akaike's Information Criterion (AIC), Akaike's Information Criterion correction (AICc), and Bayesian Information Criterion (BIC).

Additional Review

To adhere to the journal template, we have modified the figures, adjusted the caption styles, and addressed any relevant issues as needed.

General: We thank all reviewers for their constructive suggestions and comments to improve the quality of the paper in the Acknowledgment section.

Yours sincerely,

Md. Kamrujjaman

Editor-in-Chief/Academic Editor
PLOS ONE
